# Co‐adaptation impacts the robustness of predator–prey dynamics against perturbations

**DOI:** 10.1002/ece3.5006

**Published:** 2019-03-05

**Authors:** Michael Raatz, Ellen van Velzen, Ursula Gaedke

**Affiliations:** ^1^ Institute of Biochemistry and Biology University of Potsdam Potsdam Germany

**Keywords:** disturbance, evolutionary rescue, population dynamics, stability, trait adaptation

## Abstract

Global change threatens the maintenance of ecosystem functions that are shaped by the persistence and dynamics of populations. It has been shown that the persistence of species increases if they possess larger trait adaptability. Here, we investigate whether trait adaptability also affects the robustness of population dynamics of interacting species and thereby shapes the reliability of ecosystem functions that are driven by these dynamics. We model co‐adaptation in a predator–prey system as changes to predator offense and prey defense due to evolution or phenotypic plasticity. We investigate how trait adaptation affects the robustness of population dynamics against press perturbations to environmental parameters and against pulse perturbations targeting species abundances and their trait values. Robustness of population dynamics is characterized by resilience, elasticity, and resistance. In addition to employing established measures for resilience and elasticity against pulse perturbations (extinction probability and return time), we propose the warping distance as a new measure for resistance against press perturbations, which compares the shapes and amplitudes of pre‐ and post‐perturbation population dynamics. As expected, we find that the robustness of population dynamics depends on the speed of adaptation, but in nontrivial ways. Elasticity increases with speed of adaptation as the system returns more rapidly to the pre‐perturbation state. Resilience, in turn, is enhanced by intermediate speeds of adaptation, as here trait adaptation dampens biomass oscillations. The resistance of population dynamics strongly depends on the target of the press perturbation, preventing a simple relationship with the adaptation speed. In general, we find that low robustness often coincides with high amplitudes of population dynamics. Hence, amplitudes may indicate the robustness against perturbations also in other natural systems with similar dynamics. Our findings show that besides counteracting extinctions, trait adaptation indeed strongly affects the robustness of population dynamics against press and pulse perturbations.

## 1 INTRODUCTION

Environmental perturbations are likely to occur more frequently in many ecosystems as local conditions for communities are altered permanently, for example, due to climate and land‐use changes, and instantaneously, for example, by harvesting or extreme weather events (IPBES, 2018; Meehl & Tebaldi, 2004; Rahmstorf & Coumou, 2011). Population dynamics are a key determinant of many ecosystem functions (Barraquand et al., 2017; Bauer, Vos, Klauschies, & Gaedke, 2014; Lovett et al., 2002; Yang, 2004). To evaluate the stability of ecosystem functions facing global change, it is therefore of crucial importance to understand how population dynamics are impacted by such perturbations.

These population dynamics are strongly influenced by the functional traits that determine the interactions between organisms. It is by now well‐established that these traits are often flexible and may adapt to new biotic and abiotic conditions through evolution or phenotypic plasticity (Kovach‐Orr & Fussmann, 2013; West‐Eberhard, 1989). If strong enough, such adaptation may prevent extinctions of populations subject to strong environmental changes (Hughes, Inouye, Johnson, Underwood, & Vellend, 2008; Oliver et al., 2015). We hypothesize that, similar to decreasing the extinction risk, the potential for adaptation may increase the robustness of population dynamics against perturbations and thereby buffer the degree of change to the dynamics.

Prominent examples for rapid, contemporary trait adaptation are changes in offense and defense traits in predator–prey or host–parasite systems (Cortez & Weitz, 2014; Frickel, Sieber, & Becks, 2016; Kopp & Tollrian, 2003; Yoshida, Jones, Ellner, Fussmann, & Hairston, 2003). Here, trait changes increase individual fitness and can induce complex population dynamics through feedbacks between the co‐adapting offense and defense traits. Trade‐offs between the offense (defense) trait and other growth‐related traits, such as maximum growth rate, half‐saturating prey (resource) concentration, or conversion efficiency of the predator (prey), restrict an arms race and allow for cyclic trait dynamics. In such systems, organisms are torn between optimizing their fitness with respect to environmental conditions on the one hand but also with respect to the interaction between trophic levels on the other hand. Traits adapt to optimally balance between these fitness components. External perturbations could therefore have a strong impact on the population dynamics, which might, however, be mediated by trait adaptation, resulting in complex population and trait dynamics.

We study how different types of perturbations affect the dynamics of a predator–prey system and how their robustness is mediated by offense–defense co‐adaptation. Not only the type of the perturbation, but also its target (e.g., biomasses or trait values) may affect the robustness of the dynamics, that is, how the population and trait dynamics are altered by the perturbations. We therefore investigate whether the ability to adapt within ecologically relevant timescales affects the robustness of predator–prey systems and how this relationship is impacted by the perturbation type and target.

**Figure 1:**
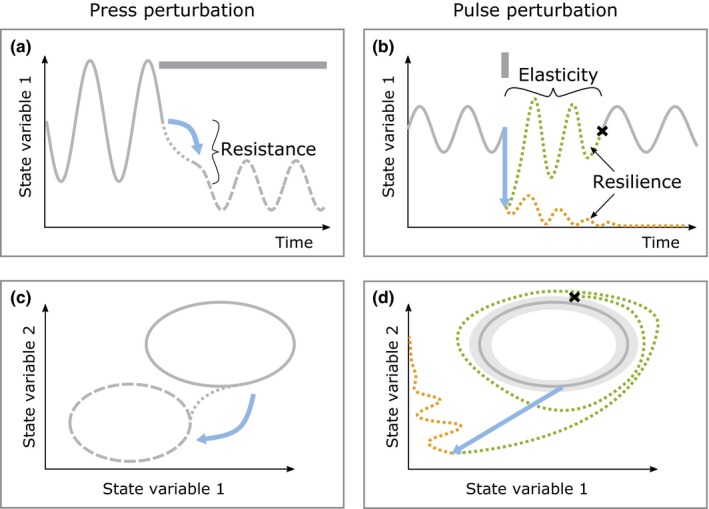
Sketch illustrating the types of perturbations and the different aspects of robustness of the population dynamics. Time series (top) and state space representations (bottom) of a system targeted by a press perturbation, that is, permanent change in a system parameter, panels (a) and (c), or a pulse perturbation, that is, an instantaneous change in one or more state variables, panels (b) and (d). Perturbations are represented by gray bars in (a) and (b), and their effect is depicted by blue arrows. A press perturbation changes the shape and location of the attractor. The distance between the pre‐ and post‐perturbation attractor (full and dashed lines in panel c) is a measure for the resistance. A pulse perturbation deflects the trajectory away from the attractor. Resilience determines whether the trajectory remains in the basin of attraction of the pre‐perturbation attractor and returns to it in a transient (dotted green line) or moves to an alternative stable state (dotted orange line). For a resilient system, elasticity determines how quickly the trajectory returns to the attractor. It is measured by the return time that passes until the trajectory enters a narrow state space volume around the attractor. This event is marked by the crosses in (b) and (d)

We will consider *press* and *pulse* perturbations as perturbation types and define corresponding robustness measures (*resistance* against press perturbations, and *resilience* and *elasticity* after pulse perturbations). Press perturbations are permanent changes to the environmental conditions, for example, by climate change, which may result in an enduring alteration of population and trait dynamics (Bender, Case, & Gilpin, 1984). We define resistance as the property that governs the magnitude of this change (Figure 1a,c; Grimm & Wissel, 1997), that is, the dynamics of a more resistant system will change less strongly. Ephemeral perturbations to the populations (e.g., by invasions or extinction events) or their traits are termed pulse perturbations (Figure 1b,d; Bender et al., 1984). Whether the system recovers from a pulse perturbation is governed by its resilience. We define elasticity as the measure for how quickly the pre‐perturbation state is reached (Grimm & Wissel, 1997). Trait adaptability may provide organisms with means to buffer the effects of press and pulse perturbations and preserve, potentially after a transient, the original population dynamics.

First, we characterize the effect of the speed of adaptation and the two most decisive environmental parameters on the dynamics themselves from bifurcation diagrams. These diagrams allow us to identify different regimes of biomass–trait dynamics along the speed of adaptation. We use the different robustness measures (resistance or resilience and elasticity) to characterize how robust these dynamics are when faced with press and pulse perturbations, respectively. We find that robustness tends to increase for faster adaptive systems, although this tendency is not exclusive but depends on further details, such as the perturbation target. From recurrent similarities between the different regimes and the robustness measures, we can infer a correlation between them and conclude that the population and trait dynamics themselves already signal their robustness against environmental perturbations. The counterintuitive nature of some of our results shows the need for detailed analyses such as ours, to guide conservation efforts and predict the impact of perturbations on the dynamics of adaptive consumer–resource systems.

## 2 METHODS

### 2.1 Model description

We investigate a predator–prey chemostat ODE model with substrate *s*, prey *x*, and predator *y*. The chemostat presents a tractable simplification of natural food webs, where the fluxes are balanced and the overall number of parameters is rather low (Smith & Waltman, 1995). Many of the numerous environmental factors that act on food webs in nature reduce to two parameters in the chemostat, the inflow concentration of substrate and the chemostat dilution rate. As a result, it is mainly these two parameters that determine the balance between bottom‐up and top‐down limitation, which is a strong determinant of the population dynamics.

Our model is parameterized for an algae–rotifer system, and the parameters are provided in Table 1. The substrate is modeled as nitrogen (μmol/L). Prey and predator are scaled to carbon (mg/L). The model reads as follows

(1) $$\begin{array}{cc} \frac{\text{d}s}{\text{d}t}= & \delta \left(s_{I}-s\right)-\frac{\omega_{N}}{\omega_{C}}r\frac{s}{K+s}x\\ \frac{\text{d}x}{\text{d}t}= & r\frac{s}{K+s}x-\frac{\mathrm{ax}}{1+ahx}y-\delta x\\ \frac{\text{d}y}{\text{d}t}= & \varepsilon \frac{\mathrm{ax}}{1+ahx}y-\delta y. \end{array}$$

**Table 1:** Parameter values and their biological meaning

Parameter	Biological meaning	Value
δ	Chemostat dilution rate^a^	0.4 day^−1^, if not stated otherwise
*s*_*I*_	Inflow resource concentration^a^	80 μmolN/L, if not stated otherwise
ω_*A*,*N*_	*N* content in an algal cell^b^	4.6 × 10^−8^μmolN
ω_*A*,*C*_	*C* content in an algal cell^b^	6 × 10^−9^mgC
*r*_0_	Maximum algal growth rate^b^	1.9 day^−1^
*K*	Algal half‐saturation^b^	49 μmolN/L
*a*_0_	Maximum attack rate of rotifersb,e	3.6/2.34 mgC^−1^ day^−1^ ≈ 1.54 mgC^−1^ day^−1^
*ɛ*_0_	Maximum conversion efficiency of rotifers^c^	1
*h*	Handling time of rotifersb,e	1/3.6 day^−1^ ≈ 0.28 day
*G*	Speed of adaptation^a^	10^−3^–10^−0.5^
*c*_*x*_	Costs for defense^c^	3
*c*_*y*_	Costs for offense^c^	2
θ	Slope of attack rate function^d^	7
*α*	Lower boundary for trait values^d^	0.001

References: ^a^Varied within this study, ^b^Raatz et al. (2018) and references therein, ^c^van Velzen and Gaedke (2017), ^d^van Velzen and Gaedke (2018), ^e^As attack rates and handling times are rarely measured, they were calculated from the predator's maximum grazing rate *g*_max_ and its half‐saturation constant *K*_Pred_ via *a* = *g*_max_/*K*_Pred_ and *h* = 1/*g*_max_.

Substrate is supplied to the chemostat system and washed out by the dilution rate δ, and *s*_*I*_ is the substrate concentration in the inflow medium. Prey growth reduces the substrate following a Monod term with maximum growth rate *r* and half‐saturation constant *K*. The growth of prey is rescaled to substrate consumption from carbon to nitrogen by the ratio of the respective per‐capita contents *ω*_*N*_/*ω*_*C*_. Prey is grazed by the predator following a Type II functional response with attack rate *a* and handling time *h*, and washed out by dilution. Predator growth is converted from grazing by the conversion efficiency *ɛ*. Washout decreases predator density and is assumed to be large compared to natural mortality, which is thus omitted for simplicity.

We assume a defense trait *u* of the prey and an offense trait *v* of the predator (both dimensionless) as in Mougi and Iwasa (2010) and van Velzen and Gaedke (2017). The difference between defense and offense determines the attack rate the predator can exert on the prey, which is implemented as a sigmoidal function (Equation (2)). The maximum slope of the attack rate function is scaled by θ. The accessible trait range is constrained by trade‐offs between prey defense *u* and maximum growth rate *r*, and predator offense *v* and conversion efficiency *ɛ*. The maximum growth rate of prey in the Monod term *r* and the conversion efficiency of predators *ɛ* decrease with increasing trait values, that is, higher defense or offense, according to Gaussians with heights *r*_0_ and *ɛ*_0_, and standard deviations 1/*c*_*x*_ and 1/*c*_*y*_. These standard deviations can be interpreted as the inverses of the costs for trait changes. For the given trait values, higher costs correspond to stronger decreases in maximum growth rate and conversion efficiency (Mougi & Iwasa, 2011; van Velzen & Gaedke, 2017).

(2) $$\begin{array}{cc} a= & \frac{a_{0}}{1+e^{\theta (u-v)}}\\ r= & r_{0}e^{-c_{x}u^{2}}\\ \varepsilon = & \varepsilon_{0}e^{-c_{y}v^{2}}. \end{array}$$

We implemented co‐adaptation between prey defense *u* and predator offense *v* following a quantitative genetics approach (Equation (3)), (Iwasa, Pomiankowski, & Nee, 1991; Mougi & Iwasa, 2010; van Velzen & Gaedke, 2017; Yamauchi & Yamamura, 2005).

(3) $$\begin{array}{c} \frac{\text{d}u}{\text{d}t}\;=\;G\frac{\partial \frac{1}{x}\frac{\text{d}x}{\text{d}t}}{\partial u}e^{-\alpha /\;u}\\ \frac{\text{d}v}{\text{d}t}\;=\;G\frac{\partial \frac{1}{y}\frac{\text{d}y}{\text{d}t}}{\partial v}e^{-\alpha /\;v}. \end{array}$$

This formulation makes no assumptions on the type of adaptation and therefore corresponds to both evolution and adaptation driven by phenotypic plasticity. Trait changes are scaled relative to the ecological dynamics by the speed of adaptation *G* (Abrams, 1992), which corresponds to the additive genetic variation in the prey and predator populations divided by their generation time in the case of evolutionary adaptation (Iwasa et al., 1991; Mougi & Iwasa, 2010, 2011). The traits change proportionally to the respective gradient of per‐capita net growth rates along the traits. The exponential functions in Equation (3) are boundary functions restricting the dynamics of *u* and *v* to positive values by decreasing the speed of trait changes when *u* or *v* approach zero (*α* = 0.001) (Abrams & Matsuda, 1997; van Velzen & Gaedke, 2017).

It has been shown that the biomass–trait dynamics in this model can be understood by examining the effective prey biomass, which is given by the product of the normalized conversion efficiency and attack rate of the predator and the prey density, ((*ɛ*/*ɛ*_0_) (*a*/*a*_0_)) *x* (van Velzen & Gaedke, 2017, 2018). This quantity informs not only about the total prey density, but also about how accessible the prey is to the predator and how efficiently it can be converted into predator growth.

The system of ordinary differential equations (Equations (1) and (3)) was integrated using the NDSolve function in Mathematica (Wolfram Research Inc. 2014). Bifurcations of the model were characterized using the continuation and bifurcation software MatCont (Dhooge, Govaerts, & Kuznetsov, 2003).

### 2.2 Obtaining a discretized attractor

The dynamics of the biomass–trait system (Equations (1) and (3)) give rise to a trajectory, i.e., a curve, that is described by the system's movement through the state space spanned by the state variables (*s*,* x*,* y*,* u*,* v*) as time passes (Figure 1). For a given parameter set, the trajectory tracks an attractor through the state space, which can be a limit cycle. The shape and size of this attractor characterize the biomass–trait dynamics and may be used to study changes to these dynamics. To allow further investigation, a discretized representation of this attractor consisting of 500 points was obtained by simulating exactly one period length of the population dynamics from initial conditions on the attractor. To determine these initial conditions, the system was simulated for 10,000 days, starting from *s*_0_ = 10 μmol/L, *x*_0_ = 1 mgC/L, *y*_0_ = 0.8 mgC/L, *u*_0_ = 0.3, *v*_0_ = 0.35. The endpoint of this simulation then served as the initial condition for the attractor. To exclude the transient, the period length was determined from the last 1,000 days of this simulation, which was discretized with a temporal resolution of 0.001 days. Using the FindPeaks function in Mathematica, the maxima in these last 1,000 days were determined. The period length was then obtained from the average time span between these maxima, excluding the edges of the observation period, which yield trivial maxima.

### 2.3 Robustness measures

#### 2.3.1 Resistance to press perturbations

Press perturbations cause permanent changes to the size, shape, and location of this attractor, by permanently changing one or more of the environmental parameters that determine the dynamics of the system. Resistance determines the sensitivity to such permanent changes. A less resistant system exhibits stronger changes for small changes in the environmental parameters. In a chemostat system, many of the natural environmental factors acting on the system can be captured by the parameters for inflow concentration *s*_*I*_ and dilution rate *δ*.

We determined the resistance of our system by assessing the dissimilarity of pre‐ and post‐perturbation attractors that arise from a permanent change in one of these two parameters (Figure 1a,c). The direction of the parameter change can be freely chosen as the dissimilarity is symmetric with respect to the choice of which attractor is the pre‐ or post‐perturbation attractor. The dissimilarity might, however, depend on the parameter region in which the perturbations occur. Thus, we created the pre‐ and postattractors from parameter changes relative to a reference parameter set *p*_*r*_ and varied this reference. The two attractors that correspond to the pre‐ and post‐perturbation states of the system *A*_1_ and *A*_2_ are then the attractors for *p*_1,2_ = (1 ± *Δ*)*p*_*r*_, where *Δ* is a small positive number giving the strength of the press perturbation. We fixed *Δ* = 0.2 in this study. Note that we do not constrain the directionality of the perturbations. Therefore, a press perturbation may decrease the parameter value from *p*_1_ = (1 + *Δ*)*p*_*r*_ to *p*_2_ = (1 − *Δ*)*p*_*r*_ or increase it from *p*_1_ = (1 − *Δ*)*p*_*r*_ to *p*_2_ = (1 + *Δ*)*p*_*r*_; both of which would result in the same pair of attractors and thus also the same dissimilarity between pre‐ and post‐perturbation attractor.

**Figure 2:**
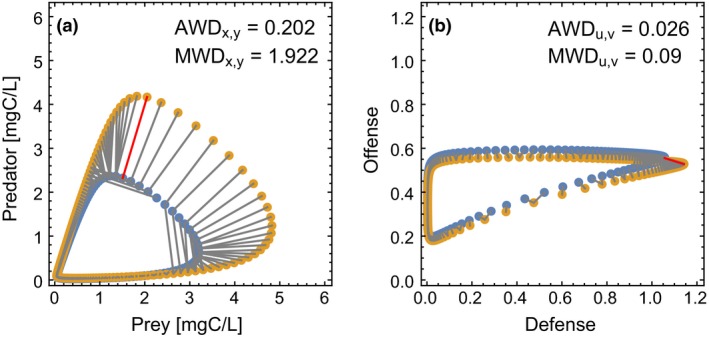
Visualization of two discretized attractors (yellow and blue dots) that correspond to pre‐ and post‐perturbation attractors of a press perturbation to the inflow concentration, *s*_*I*_ = (1 ± 0.2)*s*_*r*_, and their average and maximum warping distance (AWD and MWD) for (a) the biomass components and (b) the trait components of the attractors. Because of their different units, the distances can only be defined separately for the biomass space or the trait space. For calculating the AWD and MWD, each point on one discretized attractor is connected to its nearest point on the other attractor (gray lines). For symmetry reasons, these connections start from both attractors. Therefore, one point on one attractor can be linked to more than one point on the other attractor. The AWD is the average length of these connections, and the MWD is the length of the longest connection (red line). Parameters are *s*_*r*_ = 80 μmol/L, δ = 0.4 day^−1^ and *G* = 10^−0.5^

We developed a new method based on dynamic time warping (DTW) (Berndt & Clifford, 1994) to measure the dissimilarity between the two attractors *A*_1_ and *A*_2_. DTW allows comparing two arbitrary, open curves by warping the timescale of one curve to align it with the second curve and to detect similarities in the two curves. We used this idea and assigned each point on one attractor to its nearest point on the other attractor, thus aligning both attractors. These nearest‐neighbor links are shown in Figure 2. Note that this linking is done starting from both attractors. Thus, a point on one attractor can be linked to multiple points on the other attractor.

Using these nearest‐neighbor links, we defined two measures of attractor dissimilarity: (a) the average warping distance (AWD), which is the average of all nearest‐neighbor links, and (b) the maximum warping distance (MWD), which is the longest nearest‐neighbor link (red link in Figure 2), similar to the Fréchet distance (Eiter & Mannila, 1994; Fréchet, 1906).

The AWD averages the dissimilarities that occur along the attractors and is not strongly affected if only a few points contribute large distances; it is therefore the more conservative estimate. In contrast, the MWD increases strongly if at least one point on one attractor was displaced from the other attractor, assuming both are limit cycles. Figure 2 nicely shows that the AWD is dominated by the many small distances at smaller biomasses where the trajectories move slowly. The MWD, however, captures the short excursions in state space, which yield large distances between the attractors. Therefore, these two measures allow to discriminate between different sources of dissimilarity between two attractors and enable to detect whether perturbations alter the whole or only parts of a system's attractor.

To compute the above dissimilarity measures, a distance measure must be defined. The Euclidean distance between two points in state space can only be reasonably calculated if the units of all coordinates, that is, vector components, are equal. To compare all components of the two attractors, that is, all five dimensions (*s*,* x*,* y*,* u*, and *v*) which have different units, one way would be to rescale the attractor and set the minimum and maximum along every axis to zero and one, respectively. While doing so, we however lose the information on the size and location of the attractor, as well as change the shape strongly, both of which would render this kind of comparison little informative. Instead, we converted the substrate *s*, which is in units of nitrogen concentration, to the equivalent carbon concentration if all that nitrogen would be built into prey biomass, using the prey's carbon‐to‐nitrogen ratio. This allows us to compare substrate, prey, and predator components (all mgC/L), that is, the projections of the attractors on three dimensions from the five‐dimensional state space. Also, the traits for defense and offense have the same units (both dimensionless) and the attractor projections into trait space are directly comparable.

Using AWD and MWD, we determined the resistance of the predator–prey system in a chemostat (Equations (1) and (3)) against press perturbations to the environmental parameters of dilution rate δ and inflow concentration *s*_*I*_.

#### 2.3.2 Resilience and elasticity after pulse perturbations

Resilience and elasticity are measured after pulse perturbations to the state variables. These perturbations can target all state variables simultaneously, or individually perturb the resource, biomasses, or traits (Figure 1b,d). For example, perturbations to the substrate can be caused by flooding events or fertilization. Extinction events, for example due to draughts or fires, emigration, and immigration waves, can lead to instantaneous changes in the biomasses. Pulse perturbations to the traits can either be correlated to biomass changes or be decoupled from them. For example, an environmental cue may appear that causes instantaneous trait changes but then dissolves and releases the traits again, for example kairomones that induce zooplankton defense (Kopp & Tollrian, 2003). Accordingly, we tested different perturbation targets by perturbing either all state variables, only substrate and biomasses, or only the traits.

We simulated perturbations to the state variables by drawing random initial conditions for the differential equations (Equations (1) and (3)) from uniform distributions across reasonable intervals of the state variables (Table 2). These starting conditions represent the first time point of the trajectory after a pulse perturbation displaced it away from the attractor, which itself is not changed by the perturbation. We ran 10,000 simulations to sample the whole state space of initial conditions. Unperturbed state variables were set to a randomized location on the attractor.

**Table 2:** Boundaries for the uniform distribution from which the random initial conditions were drawn to simulate pulse perturbations

State variable	Minimum	Maximum
*s*	10^−12^ μmolN/L	80 μmolN/L
*x*	10^−12^ mgC/L	10 mgC/L
*y*	10^−12^ mgC/L	10 mgC/L
*u*	10^−12^	1
*v*	10^−12^	0.75

*Note*

The minima and maxima are set to cover the typical and reasonable ranges that the state variables attain.

Resilience, that is, whether or not a system returns to its pre‐perturbation attractor, is determined for each random perturbation by checking whether the predator biomass drops below an extinction threshold of 1 Ind/L after the perturbation. This does not account for the possibility of multistability, that is, the existence of multiple stable attractors with positive predator biomasses, which we can exclude for the parameter values investigated in this analysis (Supporting Information Figures A1 and A2 in Appendix A). Note that this method resembles empirically measuring the basin of attraction, as for example in Sanchez and Gore (2013). However, this similarity is incidental, as our model does not comprise multistability at the parameters investigated in the pulse‐perturbation analysis. The basin of attraction of the limit cycle thus covers the entire positive state space.

Elasticity is measured by the return time *t*_*r*_ that passes until the trajectory moves into a vicinity of ±3% around the pre‐perturbation attractor for the first time after the perturbation (Figure 1b,d). Perturbations which led to predator extinction were disregarded from this analysis.

The vicinity of an attractor is determined from the shortest distance *d*_CP_ between the discretized representation of attractor *A* and a point *P* of the trajectory. This distance is rescaled to the individual coordinates of the curve in state space (Equation (4)).

(4) $$d_{\mathrm{CP}}\left(A,P\right)=\min_{i\in \{0,\ldots ,n\}}\left(\sqrt{\sum_{j=1}^{m}{\left(\frac{A_{i,j}-P_{j}}{A_{i,j}}\right)}^{2}}\right)$$

Here, *n *=* *500 is the number of points on the attractor and *m *=* *5 is the number of dimensions of the system of differential equations (Equations (1) and (3)).

## 3 RESULTS

We find that the potential for trait adaptation in a predator–prey system, as characterized by the speed of adaptation (Equation (3)), strongly affects the population dynamics and three different measures for their robustness against perturbations. We quantified (a) the resistance of population dynamics against press perturbations to environmental parameters, (b) the resilience and (c) the elasticity of the system against pulse perturbations to the state variables (i.e., substrate, biomasses, and/or traits). We will start by presenting the unperturbed population and trait dynamics and then turn to how adaptation shapes the robustness of these dynamics under the impact of either press or pulse perturbations.

### 3.1 Different regimes of system dynamics

**Figure 3:**
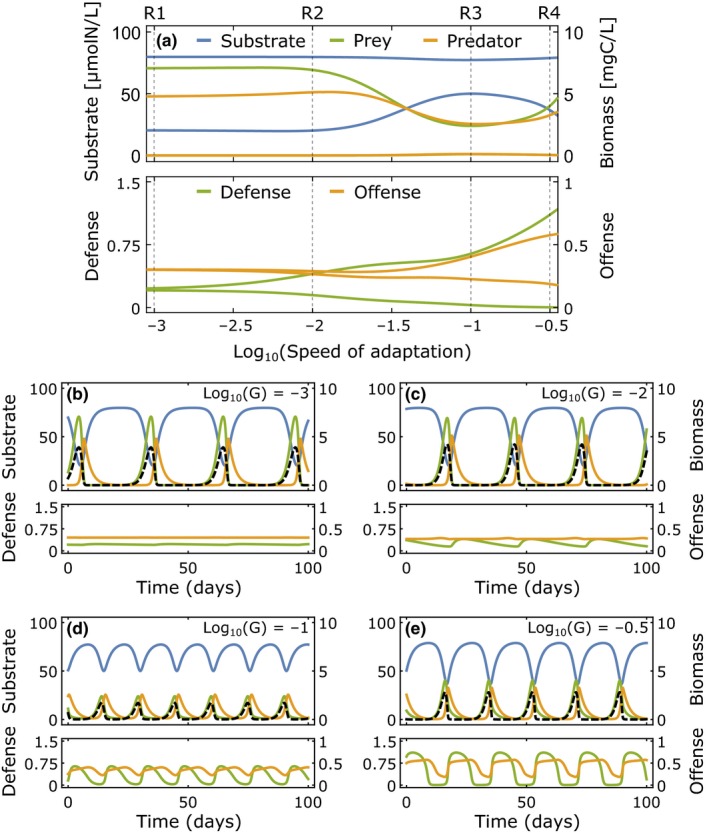
Regimes of system dynamics for an intermediate dilution rate δ = 0.4 day^−1^ and inflow concentration *s*_*I*_ **= 80 μmol/L. (a) Bifurcation diagram along the speed of adaptation *G* showing the maxima and minima of the substrate (blue), the prey and its defense (green), and the predator and its offense (orange). (b–e) System dynamics at the vertical dashed gray lines in panel (a) following the same color coding. The black dashed line shows the effective prey biomass. (b) For small *G*, mainly the biomasses oscillate (Regime R1). (c) For slightly larger *G*, the prey defense starts to show pronounced oscillations (Regime R2). (d) At larger *G*, also the offense oscillates strongly and the trait oscillations buffer the biomass oscillations (Regime R3). (e) At very high *G*, both biomasses and traits oscillate strongly (Regime R4)

We can classify four different regimes of biomass–trait dynamics (R1–R4) from the bifurcation diagram along the speed of adaptation *G* (Figure 3a). The amplitude of the trait oscillations increases for higher *G*, with the defense starting to oscillate already at lower values of *G* compared to the offense. The offense trait thus requires a higher speed of adaptation to change its value within a population cycle. The oscillation amplitudes of substrate and biomasses are large for low and high *G*, but small at intermediate speeds of adaptation. Looking closer into the dynamics, we see that for low *G*, only the predator and prey biomasses oscillate while the traits remain constant (R1) (Figure 3b). R1 therefore represents a regime where trait adaptation is negligible compared to the biomass oscillations, that is, a nonadaptive reference state. As *G* increases, this regime transits to a regime R2 where defense oscillations start to appear without significant changes in the biomass oscillations. The maxima of the effective prey biomass and therefore also the predator maxima increase slightly (Figure 3c). If *G* increases further, also the offense starts to oscillate (R3), although at smaller amplitudes than the defense. Interestingly, in this regime of trait oscillations the biomass oscillations are buffered by changes in the traits. This results in shorter oscillation periods and smaller amplitudes of predator, prey, and effective prey biomass (Figure 3d). For even higher *G*, the offense is fast enough to immediately track changes in the defense. This decreases the effect of the defense and increases the maximum effective prey biomass again, causing strong simultaneous biomass and trait oscillations (R4). Here, the basic dynamical pattern remains, but the oscillation amplitudes of all state variables increase, together with an again increasing period (Figure 3e). While their boundaries change slightly, these regimes are preserved for broad ranges of the dilution rate *δ* and inflow concentration *s*_*I*_, representing the relevant environmental factors in our system (Supporting Information Figure A3 in Appendix A).

### 3.2 Resistance of predator–prey dynamics

**Figure 4:**
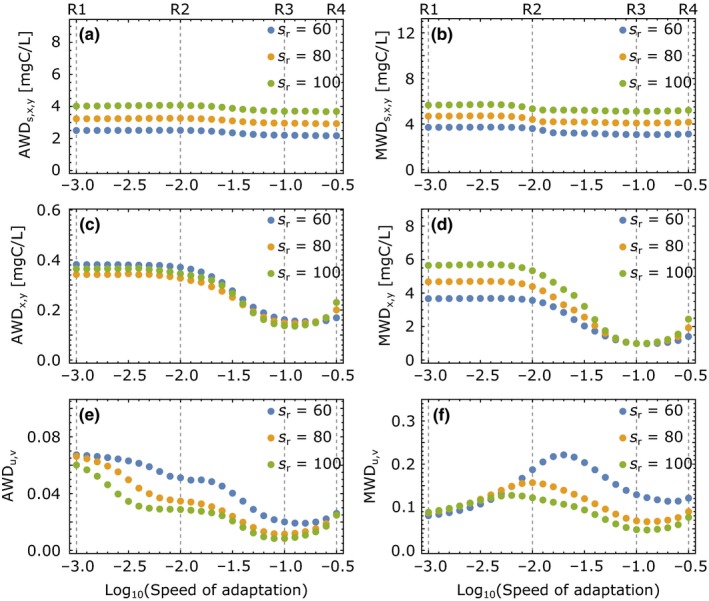
Resistance of dynamics in response to environmental press perturbations measured by the dissimilarity between the pre‐ and post‐perturbation attractors at inflow concentrations ±20% around the reference inflow concentration *s*_*r*_ (see Figure 2). (a) Average warping distance (AWD) of the attractor projection onto substrate, prey, and predator. The substrate is scaled to carbon equivalents by the prey's carbon‐to‐nitrogen ratio. (b) Maximum warping distance (MWD) of substrate, prey, and predator; (c, d) AWD and MWD between only the prey and predator components of the two attractors; (e, f) AWD and MWD between the trait components of the attractors. Smaller dissimilarities correspond to larger resistance

**Figure 5:**
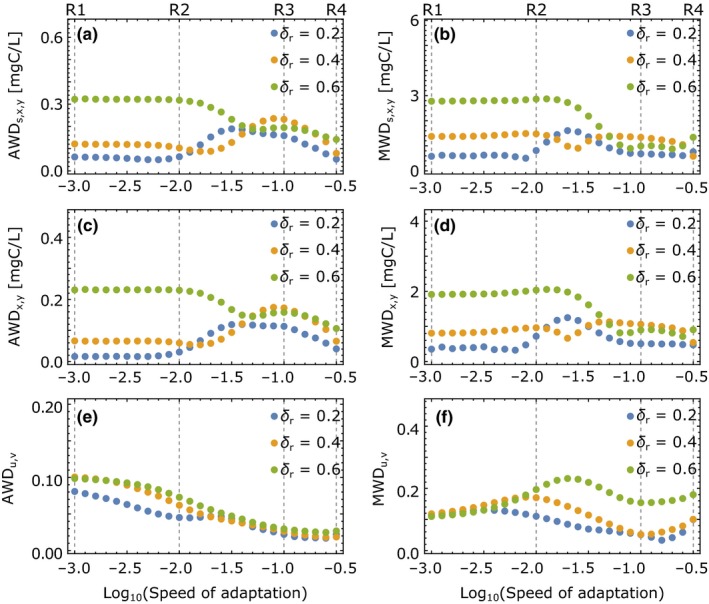
Resistance of dynamics in response to environmental press perturbations measured by the dissimilarity between two attractors at dilution rates ±20% around the reference dilution rate δ_*r*_. Further plot specifics are equal to those in Figure 4

In the following, we will quantify the resistance of the predator–prey system by measuring the dissimilarity between the pre‐ and post‐perturbation attractors which arise from a press perturbation, that is, a permanent change to either the inflow concentration or the dilution rate (Figure 1a,c). The dissimilarity is determined by comparing the two attractors at ±20% of the reference parameter value (Figures 2, 4 and 5). We employ two different measures, the average and maximum warping distance (AWD and MWD), which represent the averaged and maximum distance between the two attractors and are small for high resistance. A large AWD between pre‐ and post‐perturbation attractors indicates that the perturbation changes the dynamics over a large range of the population cycle, whereas a large MWD points to only a short, but strong delineation from the original dynamics. We find that adaptation has often strong effects on both measures, but the specific results also depend on (a) whether the inflow concentration or the dilution rate is perturbed, (b) the value of the reference parameter around which the perturbations occurs, and (c) the subset of state variables, for which the attractor dissimilarities are computed.

When the inflow concentration is perturbed, the AWD between the two attractors decreases when going from low (R1, R2) to intermediate (R3) speeds of adaptation *G*, demonstrating an enhanced resistance (Figure 4a,c,e). Increasing *G* further increases the AWD again (R4), when only biomasses or trait components of the attractors are considered. The MWD follows a similar pattern for substrate, prey, and predator (Figure 4b,d), while the traits behave differently (Figure 4f). Here, the MWD has a maximum at intermediate *G* while the AWD decreases, implying that the trait dynamics become overall more similar, but strong differences arise in a small region of the attractor.

If the dilution rate is perturbed, only the behavior of AWD and MWD of the trait components resembles the one observed for perturbations to the inflow concentration (Figure 5e,f). The patterns for dissimilarities in substrate and biomasses are instead more complex and strongly depend on the reference dilution rate. While going from R1 to R3 increases AWD and MWD for small reference dilution rates, these quantities decrease for large reference dilution rates. Increasing the speed of adaptation further from R3 to R4 then decreases AWD for all reference dilution rates. From these findings, we can conclude that the resistance of this predator–prey system tends to increase with faster adaptation if the substrate inflow is perturbed, but no simple relationship can be found if the press perturbation targets the dilution rate.

The bifurcation diagrams along the dilution rate and the inflow concentration show why the reference dilution rate has a stronger impact on the attractor dissimilarities than the value of the reference inflow concentration (Supporting Information Figures A1 and A2 in Appendix A). The AWD of two attractors at slightly different values around the reference parameter is similar to the slope of the difference between maxima and minima in these bifurcation diagrams. This slope measures how the longest axis of the attractor in one dimension changes if the environmental parameter is changed, although it does not inform about changes in attractor shape and location. If the inflow concentration increases, the predator–prey system is enriched and the minima and maxima of all state variables respond mainly monotonically with a similar behavior for different speeds of adaptation (Supporting Information Figure A1 in Appendix A). While the inflow concentration only governs the substrate input into the system, the dilution rate additionally controls the outflow, that is, the losses of substrate, prey, and predator. Therefore, the minima and maxima of substrate, prey, and predator depend non‐monotonically on the dilution rate and this dependence differs strongly for different speeds of adaptation (Supporting Information Figure A2 in Appendix A). The slope of the difference between maxima and minima therefore depends strongly on the location along the bifurcation axis, that is, the reference dilution rate, and the speed of adaptation, yielding the complex patterns found for AWD and MWD in Figure 4. Following this comparison, we also see that large slopes in bifurcation diagrams indicate low resistance to press perturbations along the bifurcation parameter as already small parameter changes induce strong changes in the state variables. From the bifurcation diagrams, we can also conclude that the speed of adaptation does not affect species coexistence along the environmental parameters that were targeted by press perturbations in our system. Nevertheless, it slightly affects the location of the Hopf bifurcation at low inflow concentrations and high dilution rates. For fast speeds of adaptation, the Hopf bifurcation becomes subcritical, creating a bistability between a fixed point and a limit cycle (Supporting Information Figure A1 in Appendix A) or between two limit cycles (Supporting Information Figure A2 in Appendix A).

### 3.3 Resilience of predator–prey dynamics

We will now present how the speed of adaptation affects the system's response to pulse perturbations (Figure 1b,d). The speed of adaptation governs whether trait adaptation can operate on ecological timescales and ranges from almost nonadaptive (R1) to highly adaptive (R4) regimes. We mimicked the pulse perturbations by setting the targeted state variables to random values drawn from a uniform distribution, thereby deflecting the system's trajectory away from the attractor.

**Figure 6:**
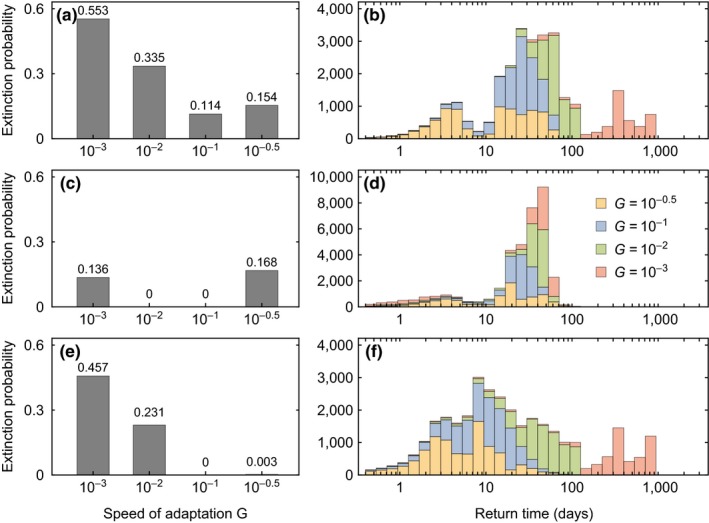
Resilience and elasticity of the predator–prey dynamics in response to random pulse perturbations, characterized by the extinction probability of the predator, that is, the proportion of simulations with predator extinction (left column), and the return time distributions of system trajectories (stacked histograms, right column). The perturbations target all state variables (panels a and b), the substrate and the biomasses (panels c and d), and the traits (panels e and f). The different speeds of adaptation *G* correspond to the four regimes of dynamics R1–R4 (Figure 3)

The resilience of the system, as characterized by the extinction probability of the predator (the proportion of simulations with predator extinction), is determined by the speed of adaptation *G*, the identity of the state variables that are targeted by the perturbation pulse, and the perturbation strength (left column in Figure 6). The highest number of extinctions occurs if all state variables are perturbed at the same time (Figure 6a). The extinction probability follows a u‐shaped trend across the speed of adaptation with a minimum at intermediate *G* = 10^−1^, corresponding to dynamics regime R3. This pattern is also present if only substrate and biomasses or only the traits are perturbed (Figure 6c,e), although the total number of extinctions is lower here.

We find that two conditions increase the likelihood of extinctions: (a) The pulse perturbation moves the system to a region of high biomasses in combination with high trait values (panels a in Supporting Information Figures A4–A6 in Appendix A), and (b) trait adaptation is either very slow (R1) or very fast (R4). The first condition, that is, a strong perturbation, moves the trajectory far away from the attractor, causing a rapid decrease in biomasses toward very small values after the perturbation, similar to an oscillator that is heavily excited. If also the trait values are high, the prey's maximum growth rate and the predator's conversion efficiency are low due to the trade‐offs, which further accelerates the biomass decline (see exemplary dynamics in Supporting Information Figure A7 in Appendix A).

If only a subset of the state variables is perturbed, the nontargeted state variables are set to a random location on the attractor. This location also affects the extinction risk, as for example choosing higher biomasses, if the traits are perturbed to high values, increases the extinction risk (Supporting Information Figure A8 in Appendix A). This pattern is further determined by the second condition, that is, higher extinction risks for nonadaptive or rapidly adaptive regimes (R1 and R4). If adaptation is slow, the traits remain at unfavorably high values and the predator goes extinct. If trait adaptation is very fast, the prey cannot decrease the grazing pressure it is exposed to by increasing its defense as the predator can directly co‐adapt and increase its offense. Thereby, the predator further decreases the prey biomass while paying the costs of a reduced conversion efficiency. Consequently, predator growth cannot compensate the losses by dilution, and the predator drives itself to extinction by co‐adapting too quickly. For intermediate speeds of adaptation, however, the biomass oscillations are buffered by trait adaptations, indicating slower rates of biomass change. This prevents strong biomass decreases during the transient after the perturbation and reduces the number of extinctions.

### 3.4 Elasticity of predator–prey dynamics

Following a pulse perturbation, the system's elasticity determines the return time, that is, the time it takes until the system trajectory arrives back in the vicinity of the attractor. For perturbations of different strength, different return times are expected, which results in a broad return time distribution. We find that these distributions are shifted toward smaller return times as the speed of adaptation increases and are in some cases bimodal (right column in Figure 6). The shift to faster returns, that is, higher elasticity, for faster adaptation is strongest if all state variables or only the traits are perturbed (panels b and f in Figure 6), which is also where the spread in return times is largest. If only substrate and biomasses are targeted (Figure 6d), the maximum return times are smaller, but still we see noticeable decreases in the return times for fast speeds of adaptation from $\bar{t_{r,\;G=10^{-3}}}$ = 35.7 days and $\bar{t_{r,\;G=10^{-2}}}$ = 37.3 days to $\bar{t_{r,\;G=10^{-1}}}$ = 24.8 days and $\bar{t_{r,\;G=10^{-0.5}}}$ = 19.7 days. This shows that even if only the biomasses of the species are changed, their trait dynamics still impact the return to the attractor.

The bimodality manifests as a clustering into faster and slower returns, with a separation around *t*_*r*_ = 10 days if only substrate and biomasses are perturbed (Figure 6d). We find that in these cases, the peak for shorter return times stems from weaker perturbations that deflected the trajectory not too far away from the attractor (panels b and c in Supporting Information Figures A4–A6 in Appendix A). Here, the trajectory returns back to the attractor already within the first transient cycle. If this is not the case, the trajectory has to perform at least another excursion through the state space before it can come closer to the attractor again and eventually penetrate the predefined 3% vicinity around the attractor.

### 3.5 Sensitivity to model assumptions

So far, we assumed the speed of adaptation to be equal for prey and predator. In Appendix B, we show that relaxing this simplifying assumption does not affect our results qualitatively. If prey adaptation is faster (or slower) than that of the predator by a factor of 2 (or 1/2), we find that the bifurcation diagrams are shifted toward smaller (larger) *G*, but they remain qualitatively unchanged (Supporting Information Figure B1 in Appendix B). Because the bifurcation plots are qualitatively unaffected, also the results for resistance are similar (Supporting Information Figures B2–B5 in Appendix B). We do find a small effect on resilience and elasticity, which both increase slightly for faster prey adaptation (Supporting Information Figures B6 and B7 in Appendix B).

Additionally, we investigated whether a different ratio between prey and predator growth rates would affect our results, as the growth rates scale both the ecological and evolutionary changes. A faster (or slower) predator growth rate could thus accelerate (decelerate) offense adaptation relative to defense adaptation. Again, we found only minor deviations from our original observations. Faster (slower) predator growth rates shift the bifurcation diagrams to smaller (larger) *G* (Supporting Information Figure B8 in Appendix B). We found that for slower predator growth rates (by approximately a factor of 2/3), the pre‐ and post‐perturbation attractors can be very different because one of the attractors may become a fixed point for low inflow concentrations or high dilution rates. This increases the dissimilarity between those attractors and decreases resistance. If both attractors remain a limit cycle, the results are again very similar to the original patterns (Supporting Information Figures B9 and B10 in Appendix B). Faster predator growth (by a factor of roughly 3/2) changes the findings for resistance only slightly (Supporting Information Figures B11 and B12 in Appendix B). Resilience was found to increase for slower predator growth with overall less extinction events and none at all if only the biomasses were perturbed (Supporting Information Figures B13 and B14 in Appendix B). Elasticity was not affected by faster or slower predator growth.

## 4 DISCUSSION

In this study, we find that trait adaptability arising from phenotypic plasticity and genetic diversity can, similar to species diversity, increase the robustness of food webs to perturbations. Earlier research has mainly focused on the effect of adaptation on species coexistence (Bell, 2017; Chevin, Lande, & Mace, 2010; Gonzalez, Ronce, Ferriere, & Hochberg, 2012; Hughes & Stachowicz, 2004; Kovach‐Orr & Fussmann, 2013; Merilä & Hendry, 2014; Oliver et al., 2015) or investigated the population dynamics of adaptive predator–prey systems without external influences (Abrams & Matsuda, 1997; Klauschies, Vasseur, & Gaedke, 2016; Mougi & Iwasa, 2010, 2011; van Velzen & Gaedke, 2017). We connected these two fields and investigated how, via inducing different regimes of population dynamics, adaptation affects robustness properties like resistance, resilience, and elasticity. We have shown that trait adaptation may increase the robustness of population dynamics of co‐adapting prey and predators against press and pulse perturbations, but that these results depend on the speed of adaptation and the type and target of the perturbation. Most importantly, our results show that the expectation of faster adaptation necessarily yielding larger robustness is not true. Instead, we find that different speeds of adaptation yield different population dynamics, which then are more or less robust against perturbations.

For press perturbations, we have seen that via affecting the population dynamics (cycle amplitude and location of the Hopf bifurcation), the speed of adaptation strongly impacts the resistance of the predator–prey system but the direction of the impact is variable. This manifests in an altered deformation and translocation of the attractor under press perturbations. For pulse perturbations, we have shown that resilience is highest in intermediately adaptive systems, as indicated by a lower extinction probability of the predator. Elasticity, that is, the speed of return back to the pre‐perturbation attractor, increased in more adaptive systems where the speed of adaptation is higher. We observed that changes in the robustness measures along the speed of adaptation coincide with changes in the amplitudes of the population dynamics that mark the transitions between different regimes of dynamics. From this, we can conclude that the population dynamics themselves already signal their robustness against perturbations, and co‐adaptation, via creating these different regimes of dynamics, therefore determines this robustness.

This relation is very apparent for the resistance of population dynamics against press perturbations. The speed of adaptation affects how the amplitude and shape of the population cycle, that is, the attractor, change along other parameters, such as the inflow concentration and the dilution rate. If these environmental parameters are targeted by press perturbations, the dissimilarities between pre‐ and post‐perturbation attractors are therefore also shaped by the speed of adaptation. A first and intuitive estimate of how the predator–prey system responds to these perturbations could be the slope of maxima and minima in bifurcation diagrams for the perturbed parameter or the slope of the amplitudes. Accordingly, we found the reason for the complex relationship between speed of adaptation and perturbation target already in these diagrams. If the amplitudes change non‐monotonically along the perturbed parameter, as for the dilution rate in our system, and if the speed of adaptation deforms these amplitudes in a complex way, it is obvious that resistance will show no simple dependence on adaptation speed. Taking a more comprehensive approach, we proposed the average warping distance AWD and the maximum warping distance MWD to measure the distance between the pre‐ and post‐perturbation attractors and quantify the resistance of the system against press perturbations. These account also for more complex changes in the shape of the attractor, which otherwise could unnoticedly become highly complex, for example, multiple local maxima of one species within one population cycle could arise (as e.g., in Raatz, Schälicke, Sieber, Wacker, & Gaedke, 2018).

Differences of AWD and MWD indicate whether perturbations have on average a large effect or whether this effect becomes pronounced only for a short period of the whole population cycle. Figure 4e,f reveals that the AWD decreases when increasing the speed of adaptation from R1 to R2, while the MWD increases. This indicates that the pre‐ and post‐perturbation attractors become more similar on average, but differences in a short region of the two attractors increase. Translated to perturbations of natural systems, this may yield different management implications. If management aims to minimize the maximum effect of perturbations, for example, an outbreak of a pest for a limited time within one vegetation period, a lower MWD may be more desirable and compensate for a higher AWD. While in our system we applied these measures mostly to limit cycles, they can also be used to quantify the translation of stable fixed points along an environmental parameter, where the AWD would equal the MWD.

The system's response to pulse perturbations is also affected by the amplitude and shape of the biomass–trait oscillations, and how these are impacted by adaptation. Here, the relationship between robustness and trait adaptation is more straightforward. If the system exhibits strong biomass oscillations already in the unperturbed state, the oscillations following a pulse perturbation will also be strong and make extinctions more likely. This explains the higher extinction risk for slow (R1) and very fast adaptation (R4). For intermediate adaptation speeds, the biomass oscillations are dampened and buffered by trait oscillations, which reduces extinctions. This dampening of biomass oscillations is caused by especially efficient prey adaptation, as prey defense can be upregulated without the predator offense immediately following in this parameter region.

If the unperturbed system shows strong trait oscillations (i.e., fast adaptation), this indicates that trait changes are possible on ecological timescales and therefore also allow the species to respond quickly to perturbations by changing their traits. Accordingly, we have seen that faster speeds of adaptation allow a faster return from changed population densities and traits back to the pre‐perturbation attractor. Mathematically, the shift toward smaller return times with larger speeds of adaptation *G* is expected as *G* directly scales the speed at which the trajectory can move through state space, at least in the coordinates of prey defense and predator offense. For small speeds of adaptation, trait dynamics are much slower than biomass dynamics and the return to the attractor is slowed down by slow changes in the traits. However, it is interesting and rather unexpected that we also find the shift toward smaller return times with increasing adaptation speed if only substrate and biomasses are perturbed. Here, a biomass–trait feedback manifests where faster trait adaptation enables the predator–prey system to respond more rapidly to instantaneous biomass perturbations. This faster return is ecologically relevant as it decreases the time the system spends in transient phases, with potentially larger cycle amplitudes, which were found to increase the extinction risk (Inchausti, Halleyt, & Halley, 2003; Pimm, Jones, & Diamond, 1988).

As stated above, the impact of co‐adaptation on predator—prey population dynamics seems to be of central importance for their robustness against perturbations. In our system, we encountered four regimes of dynamics where biomasses and trait values oscillated with different amplitudes. The transitions between these regimes coincided with changes in the robustness measures. Predator—prey systems that differ from ours might comprise only some of these regimes or have them differently ordered. Nevertheless also in these systems, the speed of adaptation should strongly affect the robustness of population dynamics, with similar dynamics—robustness correlations.

How co‐adaptation shapes different regimes of population dynamics has been investigated before (see e.g., Abrams & Matsuda, 1997; Cortez, 2018; Mougi & Iwasa, 2010, 2011; Patel, Cortez, & Schreiber, 2018; Tien & Ellner, 2012; van Velzen & Gaedke, 2017; Yamauchi & Yamamura, 2005). The particular findings, that is, whether faster trait adaptation decreases the amplitudes of species’ biomasses and increases the amplitudes of trait oscillations, are not conclusive but seem to depend on the system under investigation. Also in our system, these patterns are rather complex. Comparing the four different regimes of population dynamics shows that, as the speed of adaptation increases from slow to intermediate values, biomass oscillations decrease while trait oscillations increase. This was also observed by Mougi and Iwasa (2010), although the models differ slightly in their structure (Rosenzweig‐MacArthur vs. chemostat here), trade‐off shape (linear vs. Gaussian here), and parameterization. In contrast to their study, however, we find that biomass oscillations increase again toward even faster adaptation and occur together with pronounced trait oscillations. This overall pattern manifests in both resistance and resilience and determines the system's response to the perturbations. Interestingly, it agrees with the principle of energy flux (Rip & McCann, 2011) which states that a higher energy flux to the highest trophic level in a food chain causes stronger oscillations and is nicely captured by the effective prey biomass in our system. If mainly the defense of the prey adapts during a population cycle (R3), the energy flux to the predator decreases as the effective prey biomass is low. The effective prey biomass increases if adaptation is too slow for defense to increase effectively within a population cycle (R1 and R2), or fast enough for the offense to also be upregulated in response to an increased defense (R4). This corresponds to a higher energy flux to the predator at slow and fast speeds of adaptation and coincides with stronger biomass cycles.

Besides focussing on whether adaptation increases or decreases cycle amplitudes, or results in stable fixed points, a second line of research investigated the effect of adaptation on the stability of these fixed points (Cortez, 2018; Patel et al., 2018). Here, it was found that adaptation creates different feedbacks between biomass and trait dynamics, and the relative magnitude of these feedbacks determines whether larger adaptability stabilizes or destabilizes the fixed point. This stability ensures that any perturbation pulse to the biomasses decays in time and the system returns to the fixed point, that is, the system is resilient. Similarly, how adaptability affects resistance of fixed points against environmental press perturbations was measured by Barabás and D'Andrea (2016). They found that increased heritable adaptability increases community robustness against environmental perturbation, that is, its resistance. Often, such studies analytically compute the eigenvalues of the Jacobian at the fixed point to infer the stability of the system. Within our study, we complemented this approach and instead measured the robustness of limit cycles. Hence, we put a stronger emphasis on the actual population dynamics of prey and predator. Such cyclic dynamics are not per se less robust than fixed points and should not be viewed as less desirable ecosystem states. We thus argue that future investigations into how adaptation affects the robustness of population dynamics should take a holistic perspective considering both fixed points and limit cycles by combining analytical and simulation techniques.

Typically, the robustness of populations or ecosystems against perturbations is measured for traits that are directly linked to environmental parameters, for example, maximum critical temperatures or nutrient affinities, and adaptability of such traits to changing conditions provides obvious fitness advantages (Agashe, Falk, & Bolnick, 2011; Bell & Gonzalez, 2009; Ramsayer, Kaltz, & Hochberg, 2013). Moving beyond that, we considered trait adaptability in defense and offense, where advantages are not that obvious as these two traits are not directly related to environmental impacts but determine interspecific, that is, trophic, interactions. We showed that adaptability of such trophic traits may also strongly impact the robustness of populations and their interactions against perturbations, as accounting for all factors that impact growth and loss is key. In our system, this includes growth and washout for prey and predators and grazing for the prey. Adaptation of defense and offense allows to alter the maximum growth rate of the prey and the conversion efficiency of the predator along the trade‐offs. This provides a flexibility to optimize the fitness of both predator and prey facing perturbations, which results from adaptability in defense and offense, even if these traits are not obviously mediating environmental impacts.

To conclude, we found that trait adaptation has strong effects on the robustness of population dynamics in a predator–prey system. We have shown that varying the potential for co‐adaptation creates different regimes of biomass–trait dynamics, which are differently impacted by press and pulse perturbations. We found that faster trait adaptation (from rapid evolution or phenotypic plasticity) can increase the robustness of population dynamics against perturbations, but in some cases also achieves the exact opposite. This shows that details, such as the perturbation type, target and strength matter. As our model makes only few assumptions on the system under investigation, it is applicable to many predator–prey, but also host–parasite and other plant–herbivore systems, all of which provide often dynamic key linkages in many ecosystems.

Interestingly, the results for press perturbations, which could result from long‐term environmental changes, such as anthropogenic landscape modifications or climate change, are less conclusive than those for pulse perturbations. This strengthens the need for ecosystem‐specific investigations of the robustness of population dynamics in changing environments to allow an ecosystem management that ensures sustained ecosystem functions and stable provisioning of ecosystem services. Our study shows that co‐adaptation should be regarded a key component in this process.

## CONFLICT OF INTEREST

None declared.

## AUTHOR'S CONTRIBUTIONS

EV and UG conceived the study. MR and EV developed the model. MR performed the model analysis and wrote the first draft. All authors contributed to later versions of the manuscript.

## Data Availability

A Mathematica notebook containing the calculations and result figures is available in Appendix C in the Supporting Information.
