# Supplementary material for: Co‐adaptation impacts the robustness of predator–prey dynamics against perturbations
Source: Ecol Evol. 2019 Mar 5;9(7):3823–36. doi: 10.1002/ece3.5006 (PMC6468077; doi:10.1002/ece3.5006)
Supplement: Supplementary file 1 [file ECE3-9-3823-s001.pdf]

Appendix A - Supplementary Figures

Supplementary material to Raatz, M., van Velzen, E., and Gaedke, U. (2019). Co-adaptation impacts the robustness of predator-prey dynamics against perturbations. *Ecology and Evolution.*

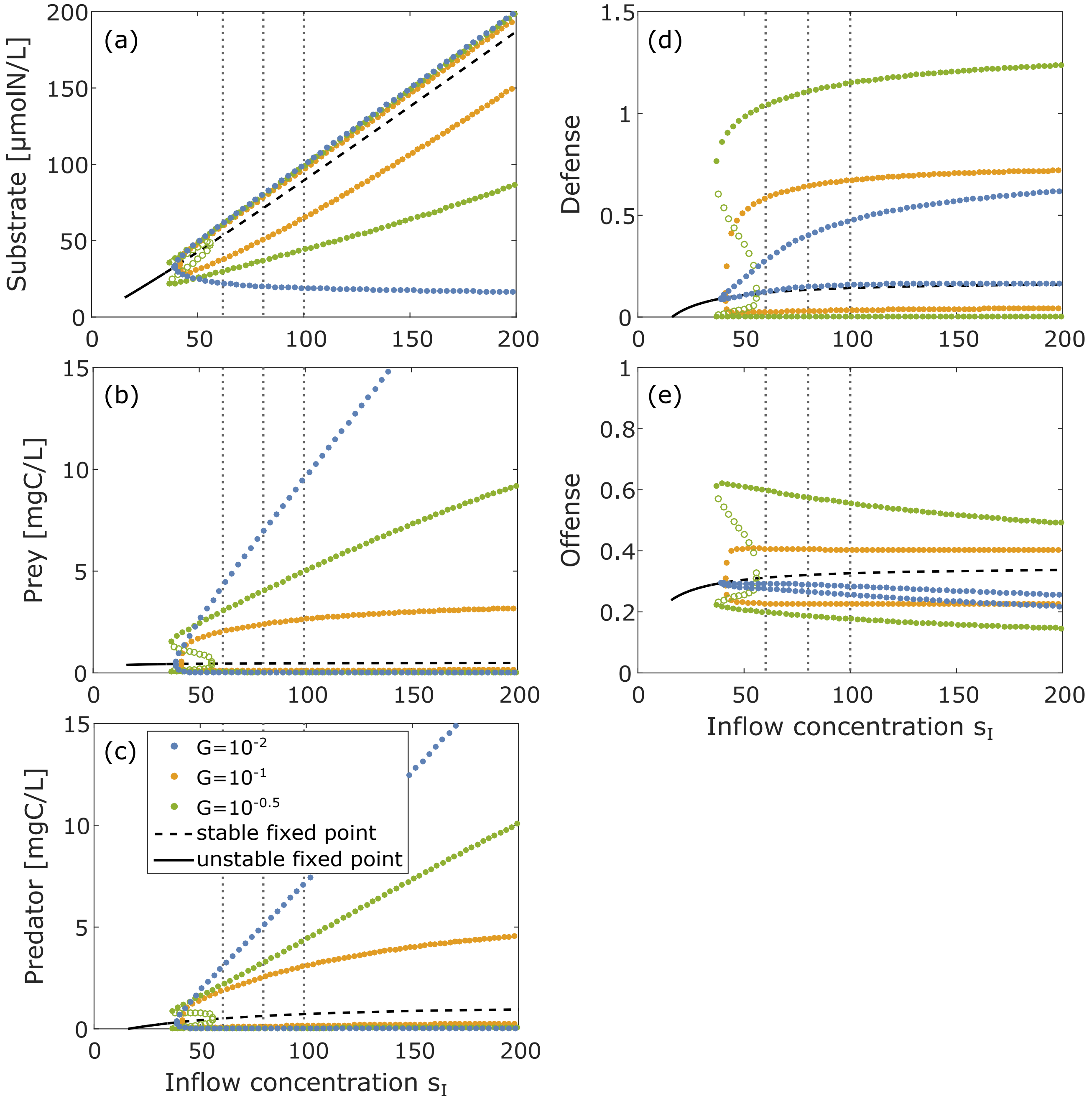

Figure A1 Bifurcation diagrams along the chemostat inflow concentration s_I as the bifurcation parameter for an intermediate dilution rate of $\delta = 0.4\,\mathrm{d}^{-1}$ for (a) substrate, (b) prey, (c) predator, (d) prey defense and (e) predator offense with different speeds of adaptation G. Dotted coloured curves show the maxima and minima of the respective state variable within a limit cycle. Closed symbols denote stable limit cycles, open symbols mark where the attractor is unstable. Stable and unstable limit cycles collide in a saddle-node bifurcation at small s_I. The black line shows the fixed point, which is unstable where the line is dashed and stable where it is continuous. Stability was confirmed by simulations. The fixed point attractor and the limit cycles are connected by a Hopf bifurcation, which is supercritical for $G = 10^{-2}$ and $G = 10^{-1}$, and subcritical for $G = 10^{-0.5}$. The location of the Hopf bifurcation along s_I changes with G. The stability of the fixed point is drawn for $G = 10^{-2}$. $G = 10^{-3}$ is omitted for clarity. Note that the combination of a subcritical Hopf bifurcation and a saddle-node bifurcation gives rise to a bistability of a fixed point and limit cycles. The dotted, vertical lines mark the reference inflow concentration s_r around which the press perturbations occur in Fig. 4 with ±20%.

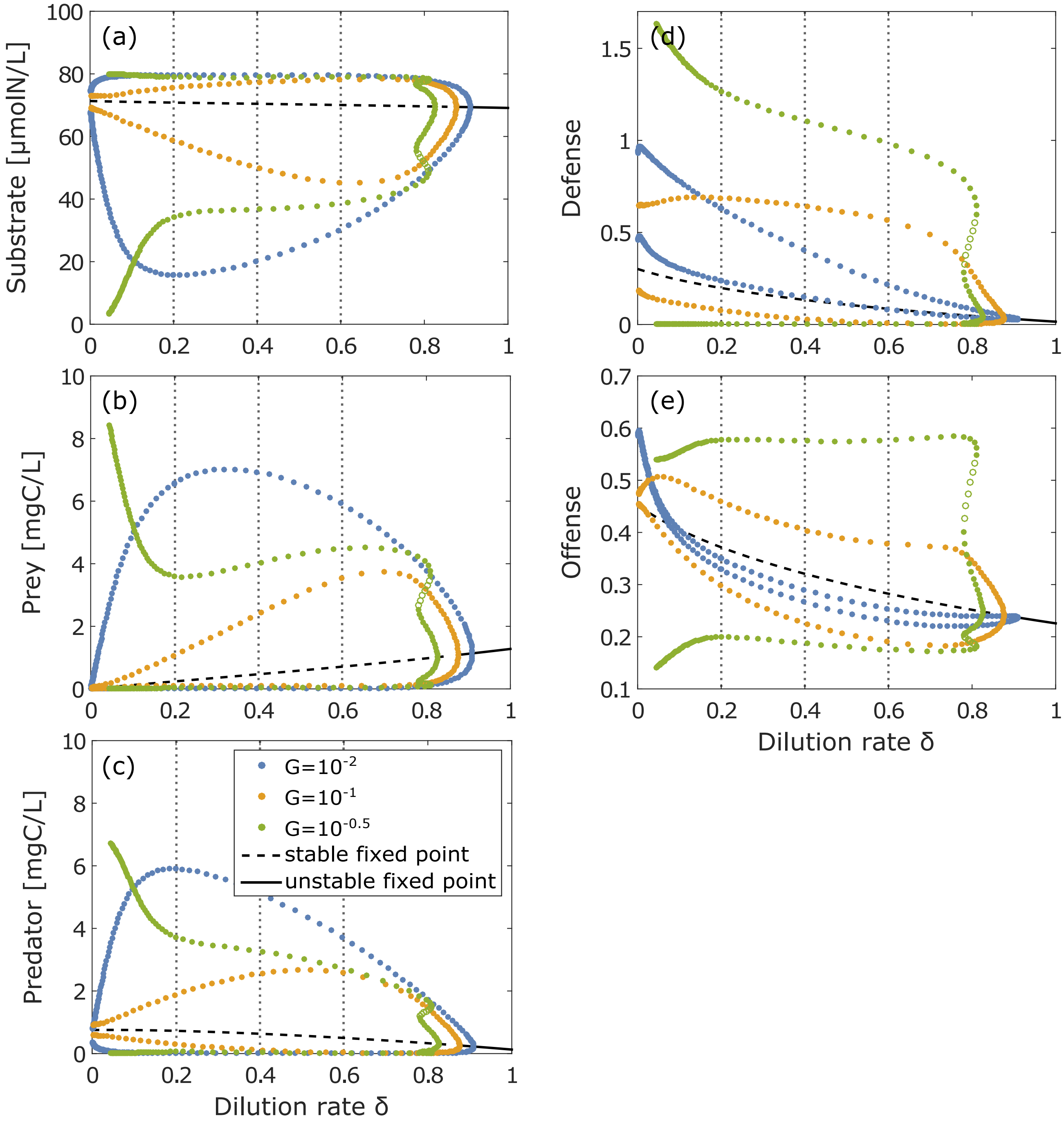

Figure A2 Bifurcation diagrams along the chemostat dilution rate δ as the bifurcation parameter for an intermediate inflow concentration of $s_I = 80\,\mu\text{mol}\,\text{L}^{-1}$. Further plot specifics as in Suppl. Fig. A1. Note that for $G = 10^{-0.5}$ we observe a Bautin bifurcation which generates a bistability of two limit cycles for large δ.

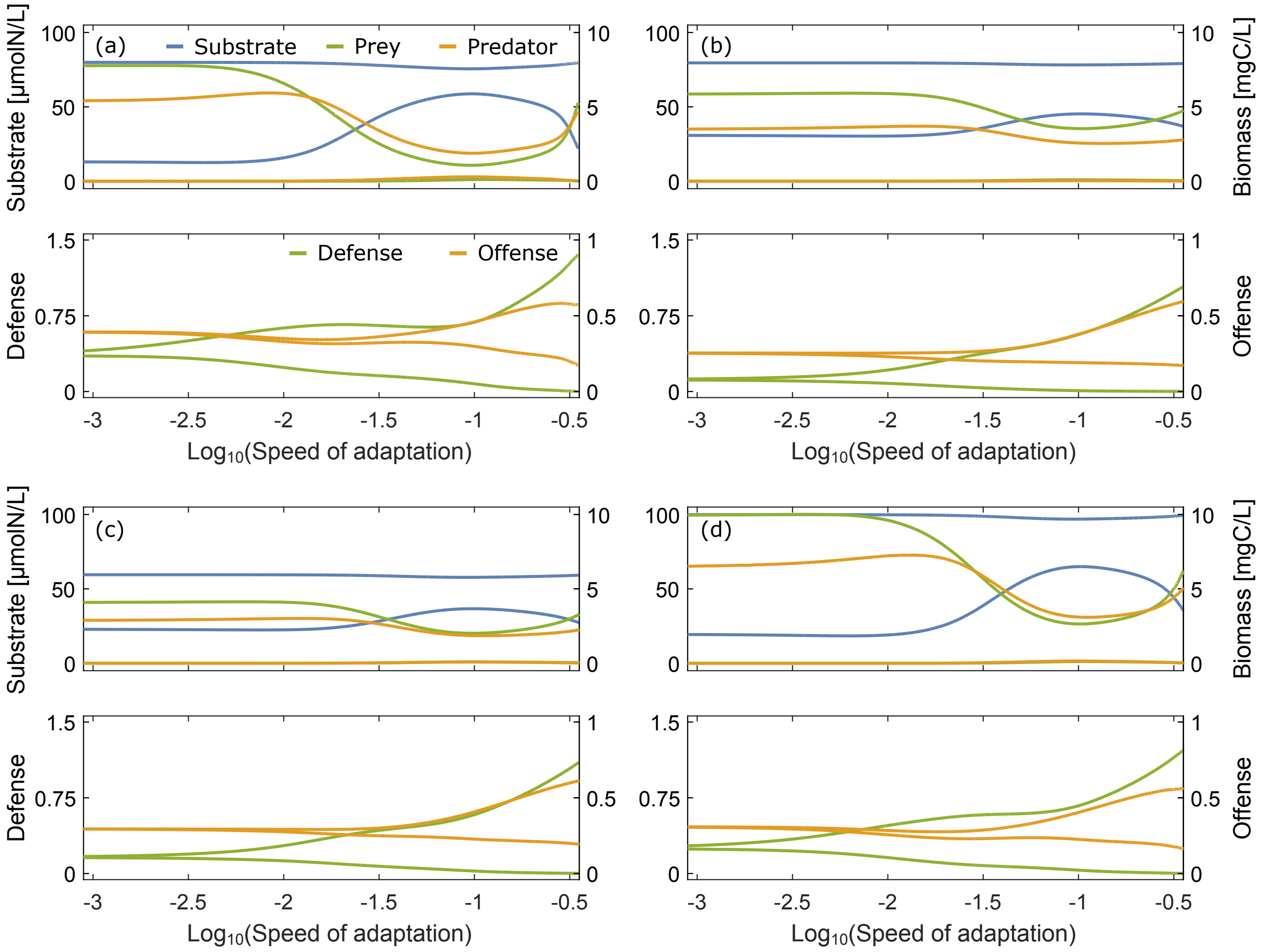

Figure A3 Bifurcation diagrams along the speed of adaptation G as the bifurcation parameter to cover different combinations of dilution rates δ and inflow concentrations s_I. Low and high dilution rates are combined with intermediate inflow concentrations and intermediate dilution rates are combined with low and high inflow concentrations, to complement Fig. 3, where both parameters are at intermediate values. (a) $\delta = 0.2\,\mathrm{d}^{-1}$ and $s_I = 80\,\mu\mathrm{mol\,L}^{-1}$, (b) $\delta = 0.6\,\mathrm{d}^{-1}$ and $s_I = 80\,\mu\mathrm{mol\,L}^{-1}$, (c) $\delta = 0.4\,\mathrm{d}^{-1}$ and $s_I = 60\,\mu\mathrm{mol\,L}^{-1}$, (d) $\delta = 0.4\,\mathrm{d}^{-1}$ and $s_I = 100\,\mu\mathrm{mol\,L}^{-1}$. The four different regimes (R1-R4) defined in Fig. 3 are apparent for all parameter combinations.

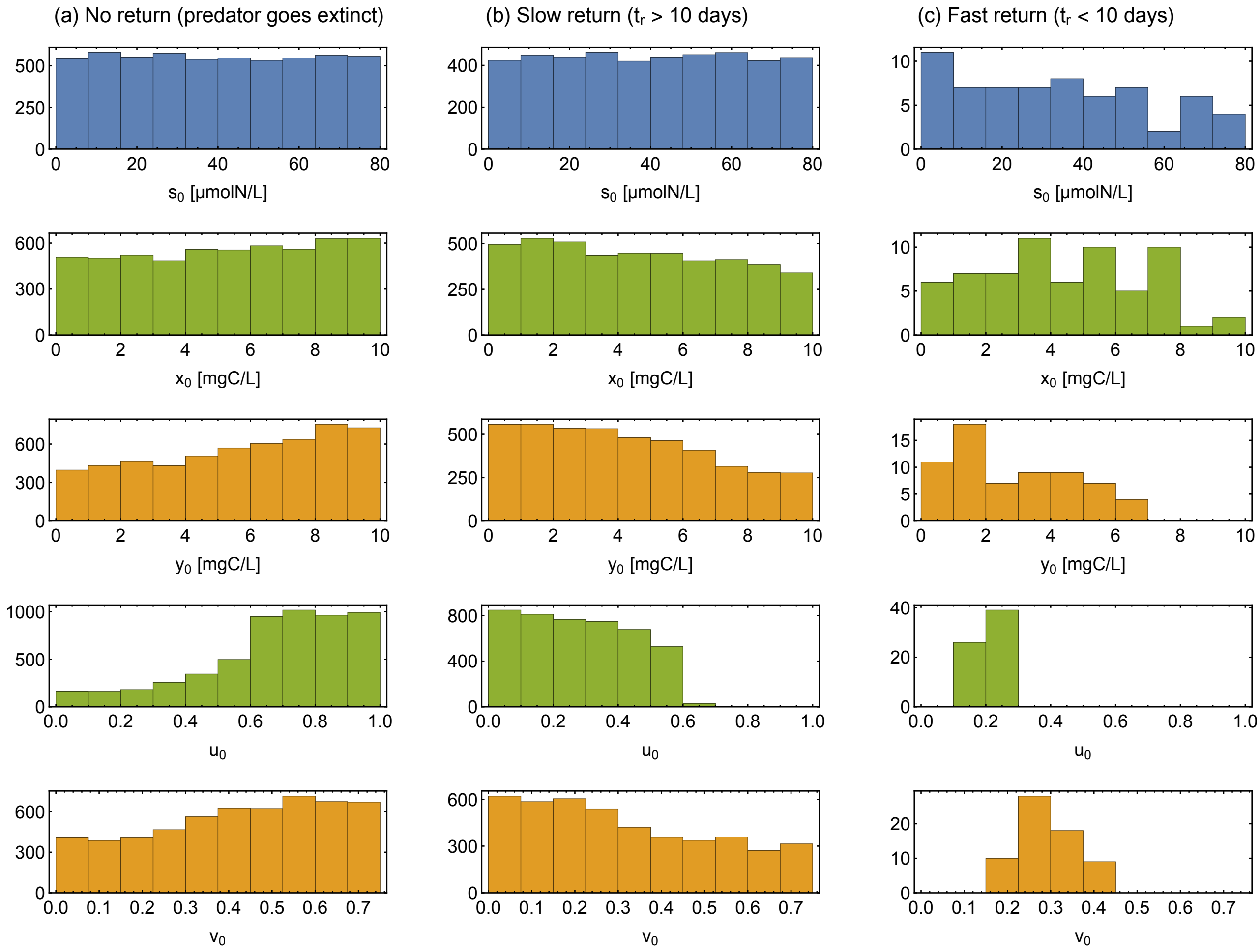

Figure A4 Histograms of the pulse perturbations, i.e. the random initial conditions for substrate s_0, prey x_0, predator y_0, defense u_0 and offense v_0, in regime R1 if all state variables are targeted, separated by extinctions (left column), slower returns (middle column) and faster returns (right column). Fast returns occur only if the traits are only slightly perturbed. Extinctions are more likely if the trajectory is perturbed to high biomasses and high trait values. Substrate perturbation is of minor importance.

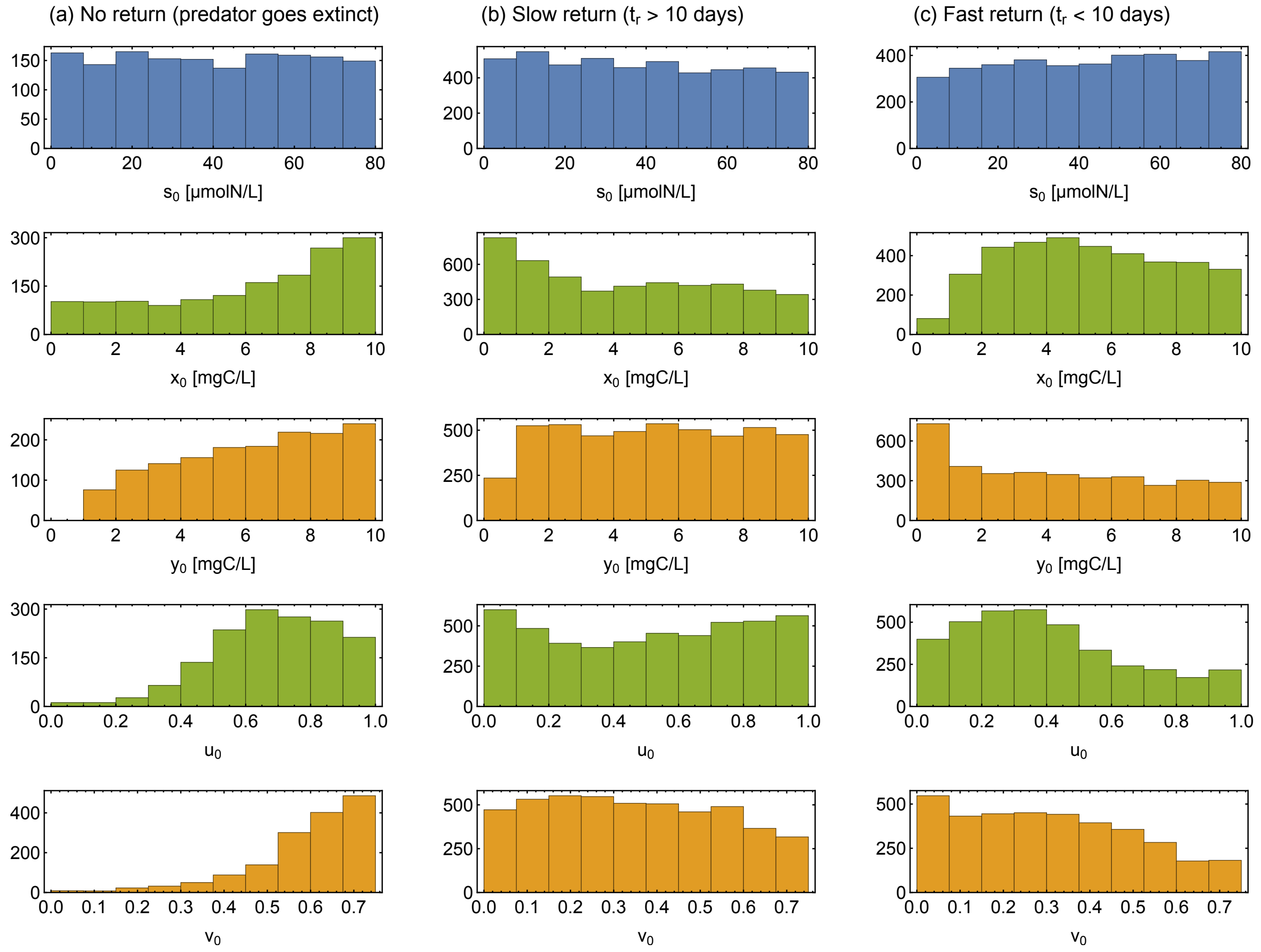

Figure A5 Histograms of the pulse perturbations, i.e. the random initial conditions, in regime R4 if all state variables are targeted. Further specifics as in Suppl. Fig. A4. Also here, faster returns are more likely for small perturbations. Extinctions are more likely if the trajectory is perturbed to high biomasses and high trait values.

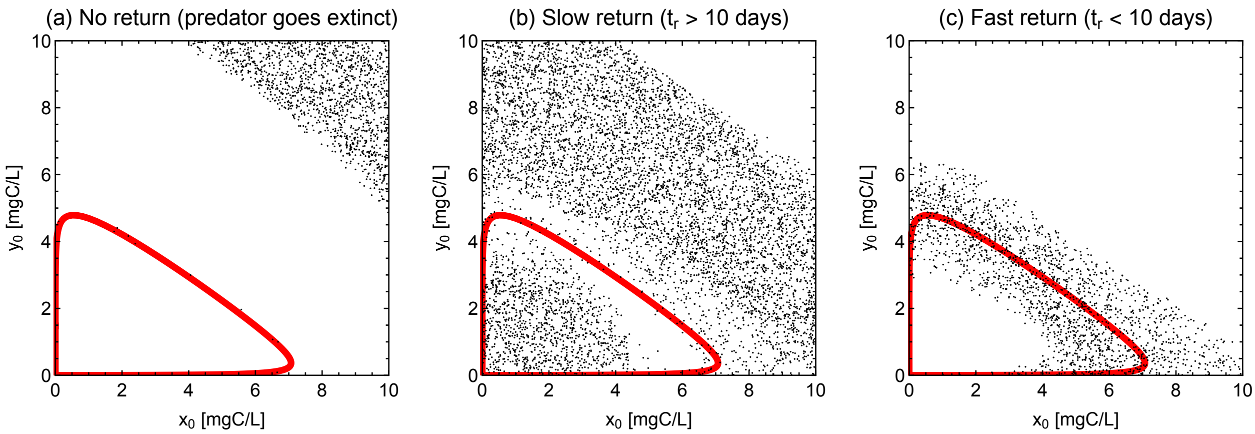

Figure A6 Scatter plots of the perturbed prey and predator biomasses x_0 and y_0 from pulse perturbations to substrate, prey and predator at $G = 10^{-3}$ (Fig. 6c-d). The respective attractor is shown in red. The perturbation results in either (a) predator extinction, (b) large or (c) small return times t_r. Extinctions occur if the trajectory is perturbed to high biomasses. Returns are faster if the displacement from the attractor is small.

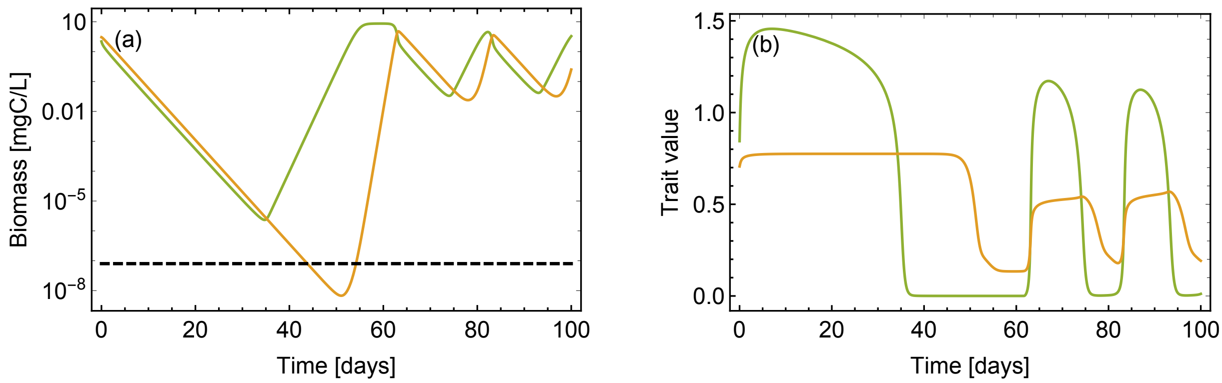

Figure A7 Dynamics of an exemplary extinction event after a pulse perturbation to the traits ($u_0 = 0.844$ and $v_0 = 0.706$) at $G = 10^{-0.5}$, showing the transient after the perturbation. The initial conditions of the biomasses, which are not targeted by the perturbation, are marked in black in Suppl. Fig. A8. (a) Prey (green) and predator biomasses (orange) and (b) defense (green) and offense (orange) move far away from the attractor following the perturbation. Eventually, the predator biomass drops below the black dashed line in (a) marking the extinction treshold.

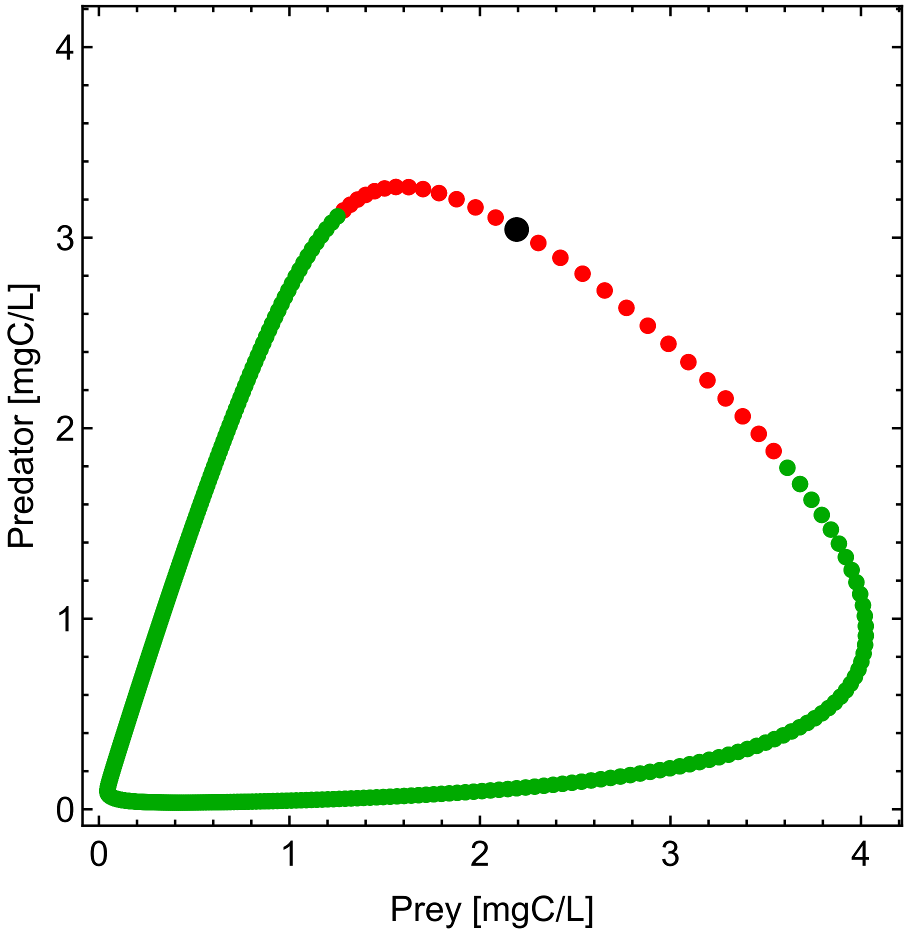

Figure A8 Effect of the choice of the position of non-targeted state variables on the attractor on the extinction risk following a pulse perturbation to a subset of the state variables. Here, defense and offense were perturbed to $u_0 = 0.844$ and $v_0 = 0.706$ at $G = 10^{-0.5}$. Green points mark choices for the biomasses where the predator persists, for red points it goes extinct. The transient dynamics for the combination marked by a black point are shown in Suppl. Fig. A7.
