# Supplementary material for: Co‐adaptation impacts the robustness of predator–prey dynamics against perturbations
Source: Ecol Evol. 2019 Mar 5;9(7):3823–36. doi: 10.1002/ece3.5006 (PMC6468077; doi:10.1002/ece3.5006)
Supplement: Supplementary file 2 [file ECE3-9-3823-s002.pdf]

Appendix B - Sensitivity analysis

Supplementary material to Raatz, M., van Velzen, E., and Gaedke, U. (2019). Co-adaptation impacts the robustness of predator-prey dynamics against perturbations. *Ecology and Evolution.*

Different speeds of adaptation for prey and predator

In the main results, we assumed that prey and predator possess the same speed of adaptation G. The speed of adaptation corresponds to the adaptive genetic variance. It might thus be quite different for particular prey and predator species. In the following, we will however show that relaxing this simplifying assumption does not qualitatively affect our results.

For this purpose, we introduce a factor that scales the rate of trait change in the prey, but keep $G_{Pred} = G$ fixed. We see that the bifurcation diagram along the speed of adaptation is shifted to the right (larger G) if prey trait adaptation is slowed down by a factor of 0.5, i.e. $G_{Prey} = G/2$, as now even higher speeds of adaptation are required for the prey to increase its defense within a population cycle (Fig. B1a). Conversely, if prey trait adaptation is sped up by a factor of two, i.e. $G_{Prey} = 2G$, considerable defense oscillations already occur at much lower speeds of adaptation and the bifurcation diagram is accordingly shifted to the left (Fig. B1b).

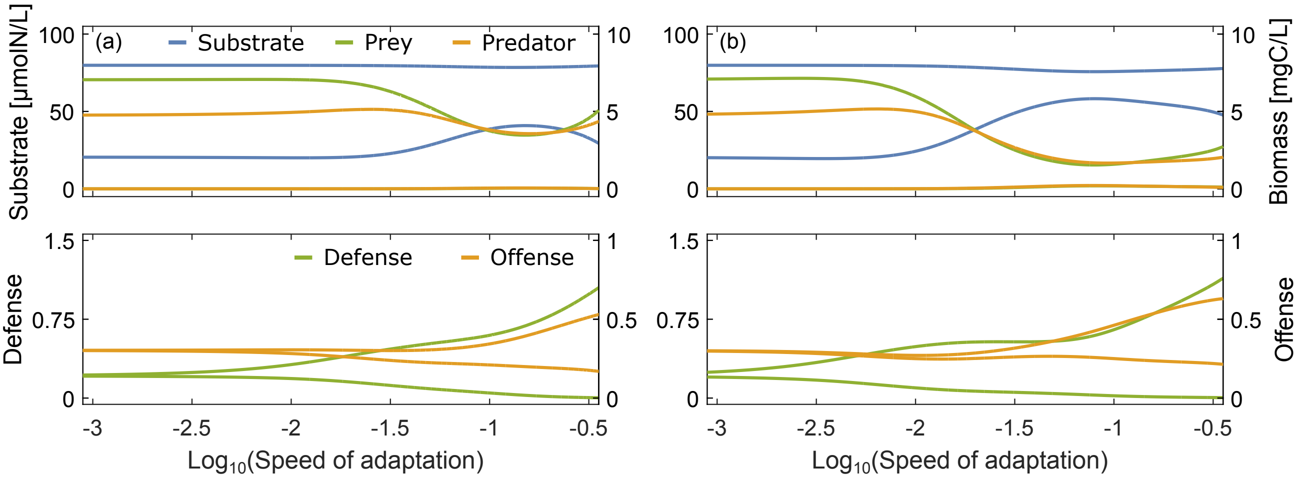

Figure B1 Bifurcation diagrams along the speed of adaptation G showing the effect of relaxing the assumption of equal adapation speed for prey and predator. (a) The rate of prey trait change is halved, i.e. the speed of prey adaptation is $G_{Prey} = G/2$ and $G_{Pred} = G$. (b) The rate of prey trait change is doubled, i.e. the speed of prey adaptation is $G_{Prey} = 2G$ and $G_{Pred} = G$.

As the overall dynamics are unchanged, also the AWD and MWD are not qualitatively affected by assuming a different speed of adaptation for prey and predator, but shifted towards higher (lower) speeds of adaptation for slower (faster) prey trait adaptation (Fig. B2-B5). The extinction probabilities

and return time distributions also remain qualitatively unaffected (Fig. B6, B7). Here we find that faster prey adaptation decreases the number of extinctions and shifts return time distributions slightly towards smaller values.

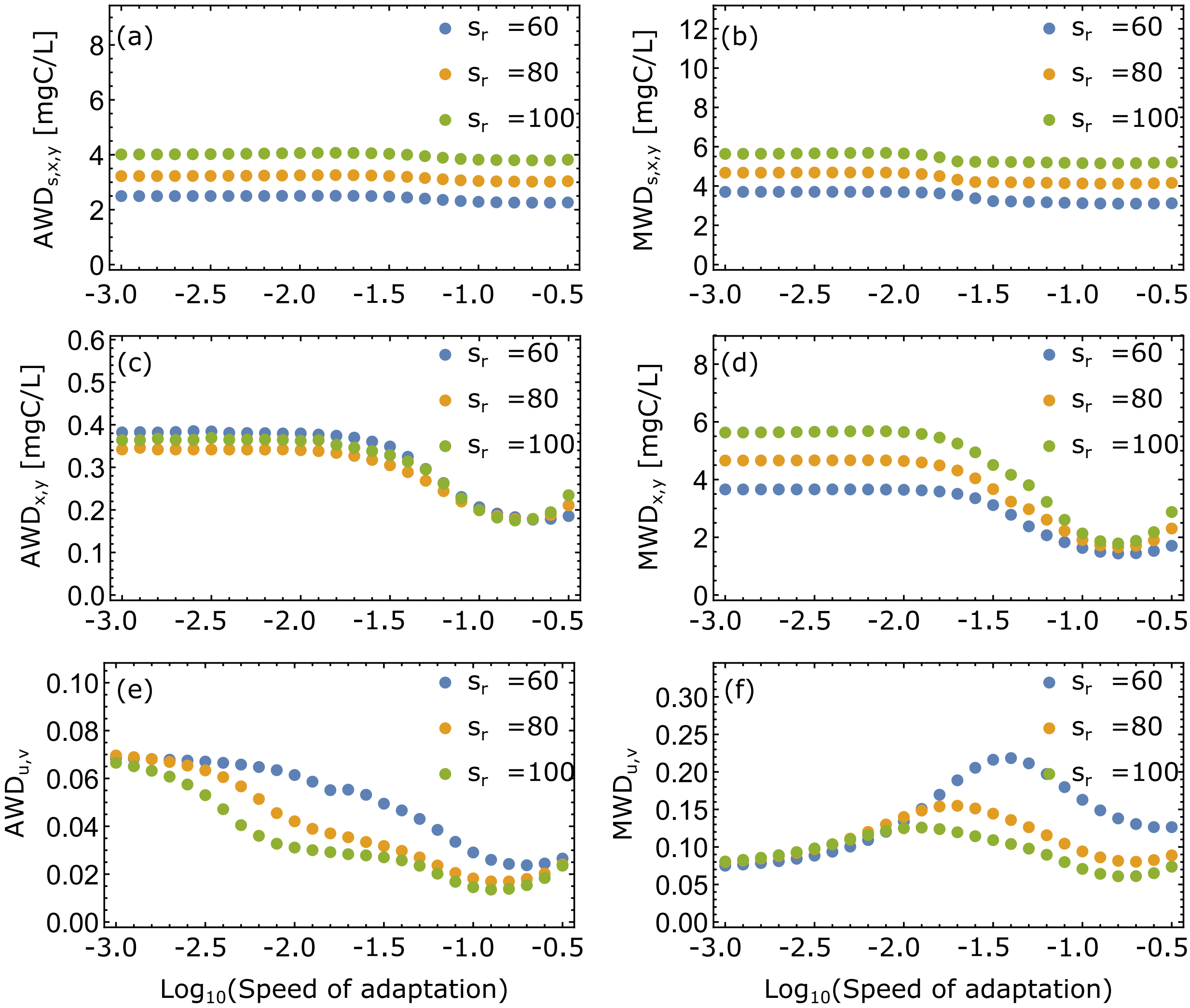

Figure B2 Resistance of dynamics in response to environmental press perturbations acting on the inflow concentration s_I for $G_{Prey} = G/2$. Further plot specifics are identical to Fig. 4.

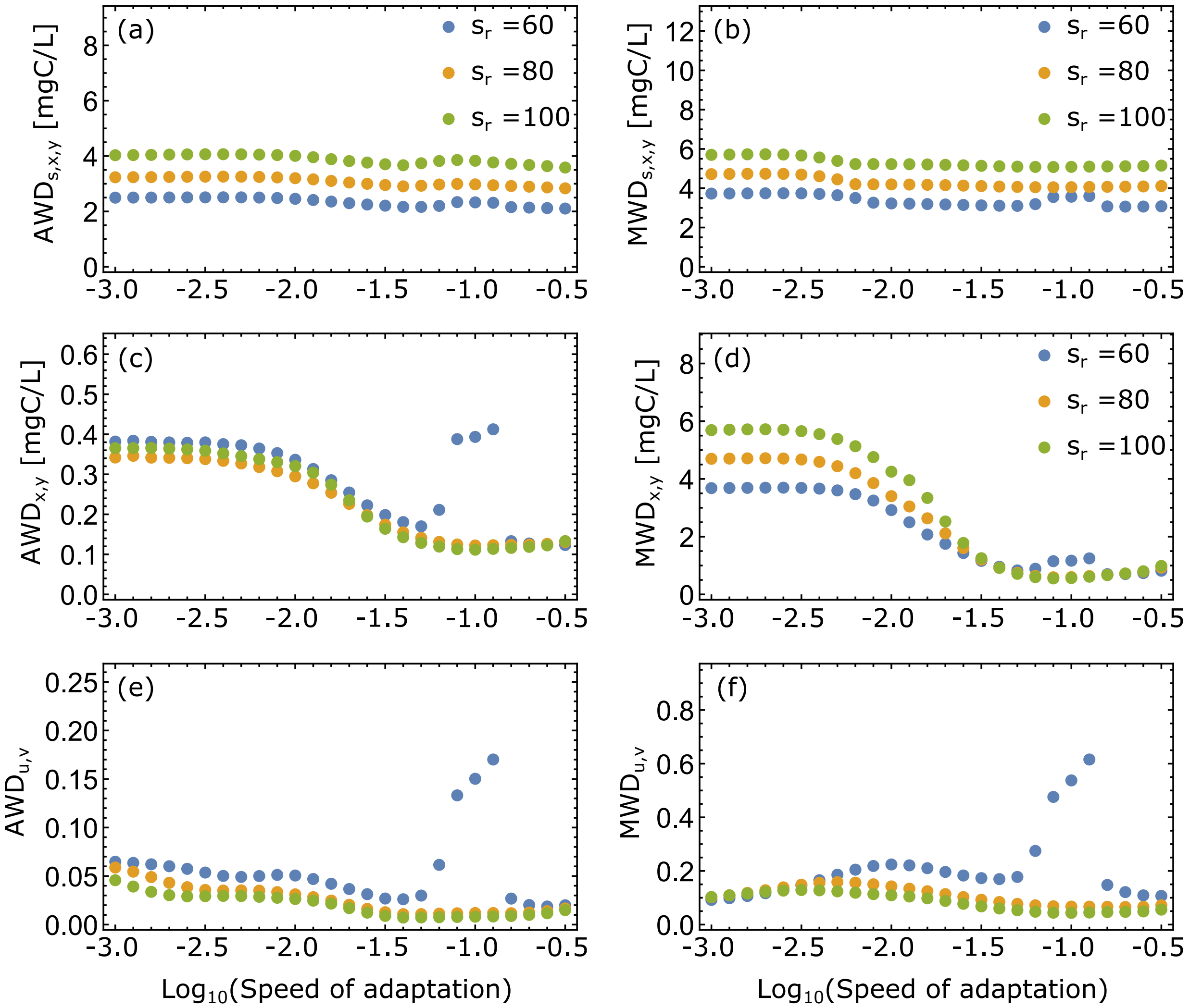

Figure B3 Resistance of dynamics in response to environmental press perturbations acting on the inflow concentration s_I for $G_{Prey} = 2G$. Further plot specifics are identical to Fig. 4. For $s_r = 60\,\mu\text{mol}\,\text{L}^{-1}$ the AWD and MWD jump to high values around $G = 0.1$ as here for $s_I = (1-0.2)\,s_r$ the system reaches a steady state, but for $s_I = (1+0.2)\,s_r$ it is cycling, causing a high dissimilarity of the two attractors.

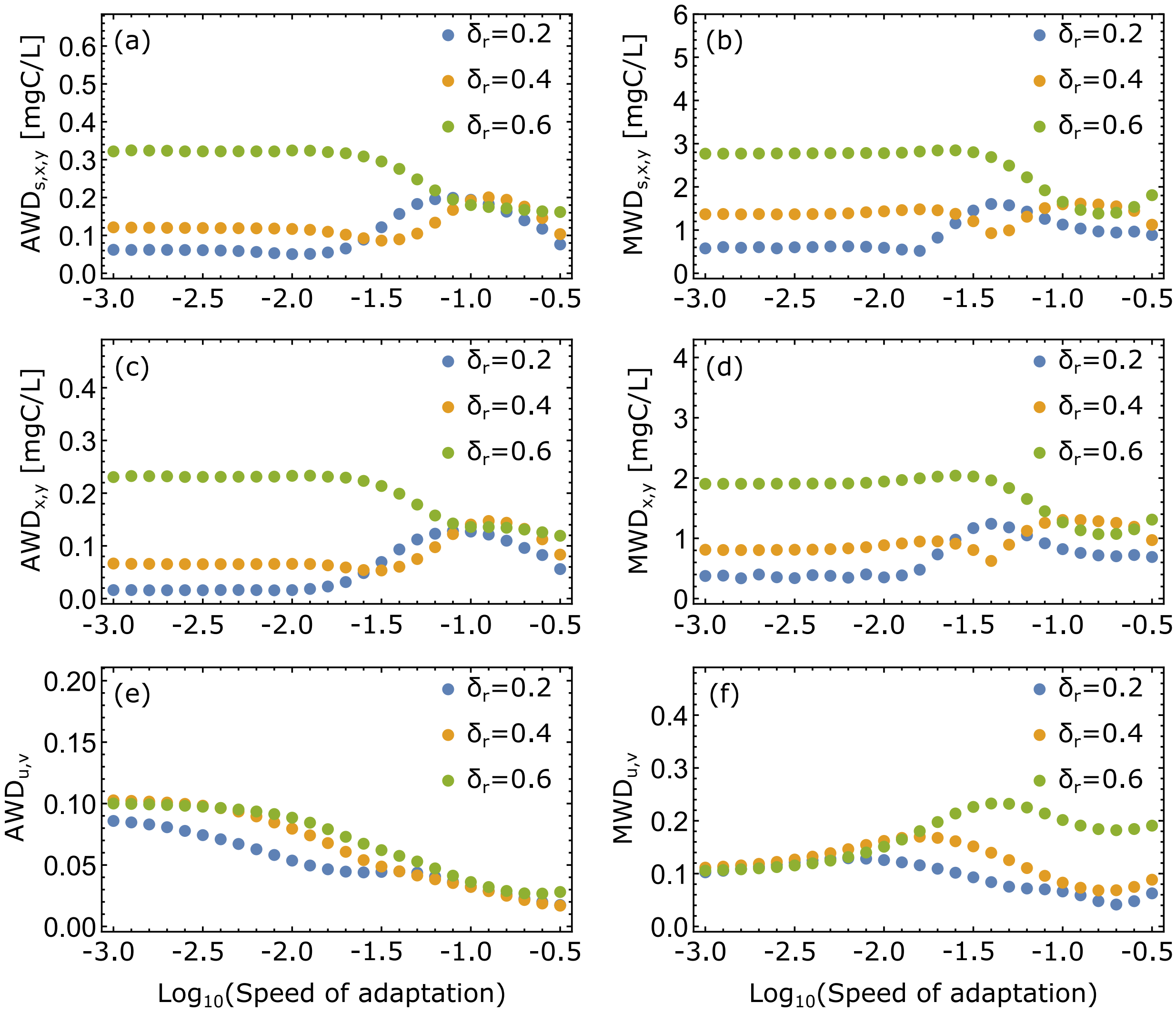

Figure B4 Resistance of dynamics in response to environmental press perturbations acting on the dilution rate δ for $G_{Prey} = G/2$. Further plot specifics are identical to Fig. 4.

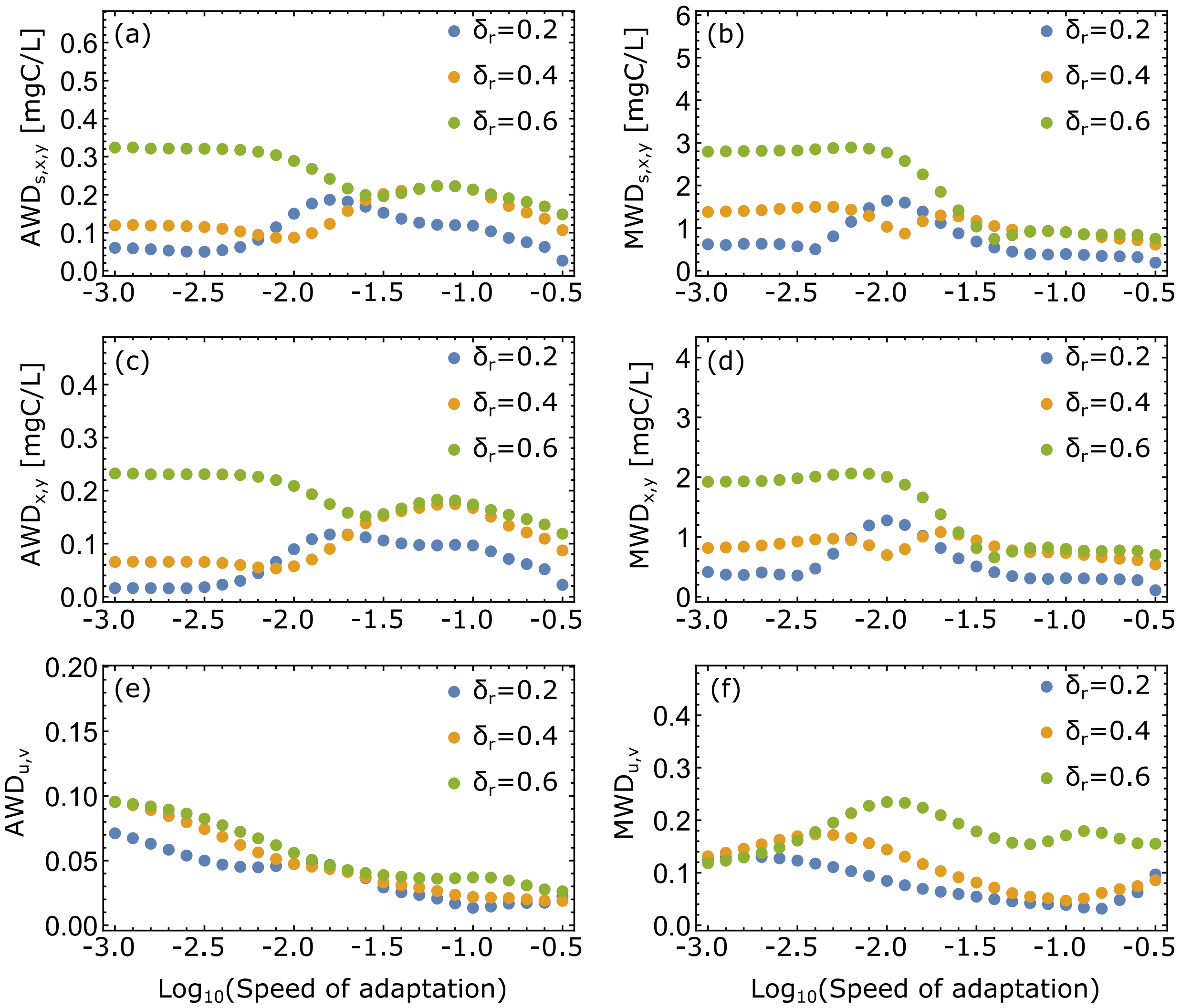

Figure B5 Resistance of dynamics in response to environmental press perturbations acting on the dilution rate δ for $G_{Prey} = 2G$. Further plot specifics are identical to Fig. 4.

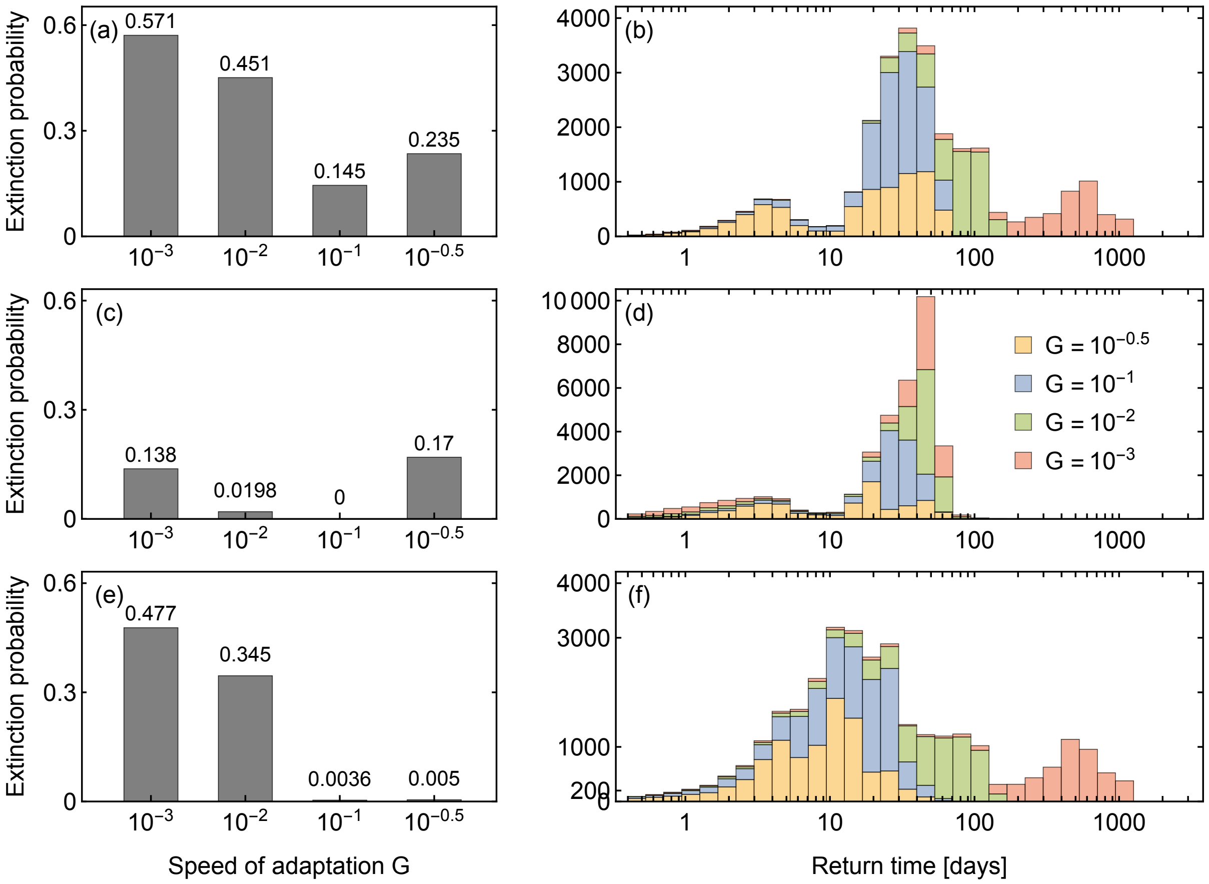

Figure B6 Resilience and elasticity of the predator-prey dynamics in response to random pulse perturbations for $G_{Prey} = G/2$. Further plot specifics are identical to Fig. 6.

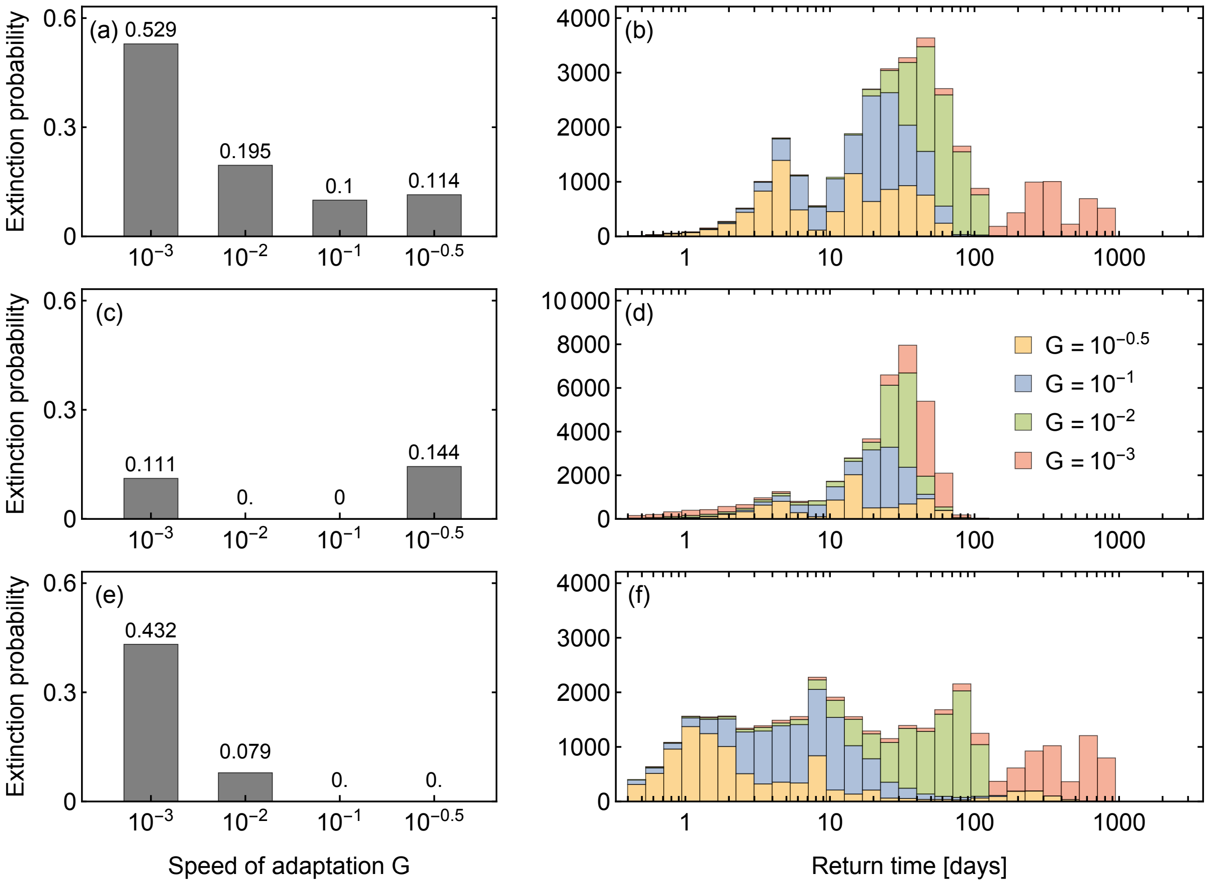

Figure B7 Resilience and elasticity of the predator-prey dynamics in response to random pulse perturbations for $G_{Prey} = 2G$. Further plot specifics are identical to Fig. 6.

Sensitivity to the predator maximum grazing rate

The maximum per-capita growth rate of the predator determines how quickly the offense trait changes (Eq. 3). It can be obtained by multiplying the maximum grazing rate of the predator g_{max} with its conversion efficiency ε, which is given in Eq. 2. The maximum grazing rate parameter thus not only scales the predator's growth rate, but also the speed of offense adaptation. Thus, a different maximum grazing rate could alter the relative speed of defense and offense adaptation. We therefore compared the results for lower or higher maximum grazing rates to our original results for $g_{max} = 3.6\,\mathrm{d}^{-1}$.

Translating the maximum grazing rate into attack rates or handling times can be achieved by equating the formulations of the predator's functional response in terms of attack rate a and handling times h (as in Eq. 1), and in terms of maximum grazing rate g_{max} and half-saturation prey density K_{Pred}. This results in

$$\frac{ax}{1+ahx} = g_{max}\frac{x}{K_{Pred}+x}\,, \tag{B1}$$

which is satisfied for $a = \frac{g_{max}}{K_{Pred}}$ and $h = \frac{1}{g_{max}}$. For our original parameter set this yielded an intermediate attack rate of $a = \frac{3.6}{2.34}\mathrm{mgC}^{-1}\,\mathrm{d}^{-1} \approx 1.54\,\mathrm{mgC}^{-1}\,\mathrm{d}^{-1}$ and an intermediate handling time of $h = \frac{1}{3.6\,\mathrm{d}^{-1}} \approx 0.28\,\mathrm{d}$.

We assumed a lowered maximum grazing rate of $g_{max} = 1.9\,\mathrm{d}^{-1}$, which equals the nutrient uptake rate of the prey. This results in a lower attack rate $a = \frac{g_{max}}{K_{Pred}} = \frac{1.9}{2.34}\mathrm{mgC}^{-1}\,\mathrm{d}^{-1} \approx 0.81\,\mathrm{mgC}^{-1}\,\mathrm{d}^{-1}$ and longer handling time $h = \frac{1}{g_{max}} = \frac{1}{1.9\,\mathrm{d}^{-1}} \approx 0.52\,\mathrm{d}$. The higher maximum grazing rate we assumed to be threefold the lower extreme value ($g_{max} = 5.7\,\mathrm{d}^{-1}$), which approximately centers the original value of $g_{max} = 3.6\,\mathrm{d}^{-1}$ between these two extremes. This results in a larger attack rate $a = \frac{5.7}{2.34}\mathrm{mgC}^{-1}\,\mathrm{d}^{-1} \approx 2.44\,\mathrm{mgC}^{-1}\,\mathrm{d}^{-1}$ and a shorter handling time $h = \frac{1}{5.7\,\mathrm{d}^{-1}} \approx 0.18\,\mathrm{d}$ for the scenario of accelerated predator grazing.

We find that smaller maximum grazing rates shift the bifurcation diagrams along the speed of adaptation G to the right, i.e. to larger G, decreasing the amplitudes of biomass oscillations for low G.

A higher g_{max} increases the maximum per-capita growth rate of the predator, enabling a faster offense adaptation for smaller G. This shifts the bifurcation diagrams to the left (Fig. B8), similar to the results for faster prey adaptation speed (Fig. B1). Also, a higher maximum grazing rate increases the predation pressure on the prey, thus driving stronger defense adaptation for smaller G. The resulting trait oscillations buffer the biomass oscillations and decrease the amplitudes of biomasses for faster speeds of adaptation.

The resistance measures are only slightly affected by the altered maximum grazing rate (Fig. B9-

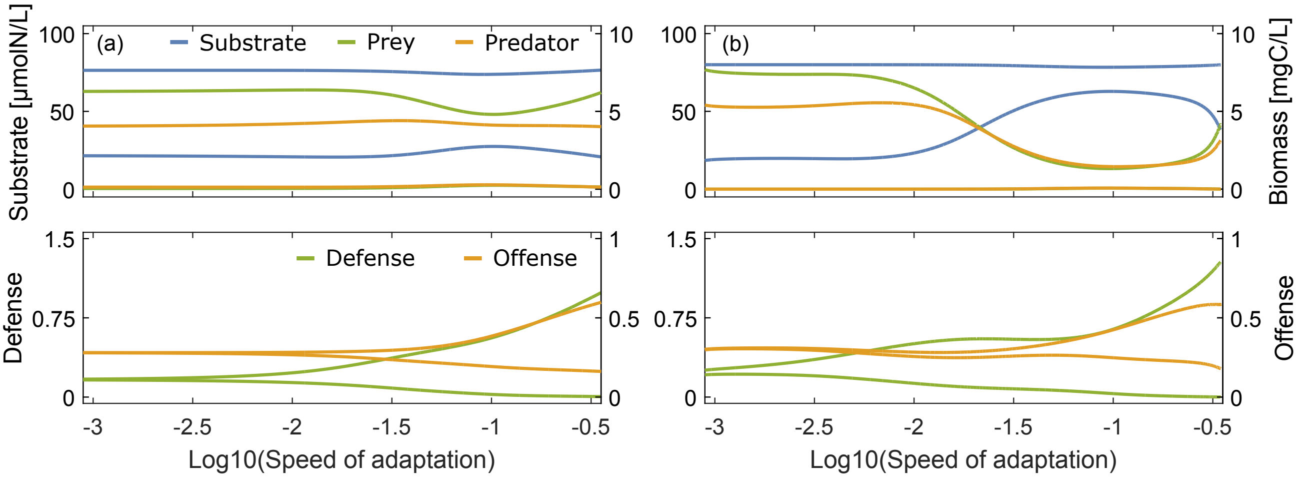

Figure B8 Bifurcation diagrams along the speed of adaptation G for (a) lower ($g_{max} = 1.9\,\mathrm{d}^{-1}$) and (b) larger ($g_{max} = 5.7\,\mathrm{d}^{-1}$) maximum grazing rate of the predator, resulting in different attack rates and handling times (see text).

B12). The most prominent difference to an intermediate maximum grazing rate occurs for slower g_{max}. Here, the AWD and MWD increase for larger speeds of adaptation, if either the reference inflow concentration is low or the reference dilution rate is high (Fig. B9, blue dots; B10, green dots). A lower inflow concentration or a higher dilution rate cause the system to reach a steady state (Fig. A1, A2). Thus, for slow predator grazing the perturbed attractors with either $s_I = (1-0.2)\,s_r$ or $\delta = (1+0.2)\,\delta_r$ are fixed points. Here, trait adaptation is zero in our model and the state variables at the fixed point are independent of the speed of adaptation. The limit cycle amplitudes of the respective other attractors increase for larger speeds of adaptation (Fig. B8), thus increasing the dissimilarity between the fixed points and the limit cycles with larger G, yielding increasing AWD and MWD. This increase is especially strong for the dissimilarities of the trait components (panels e and f in Figs. B10 and B9), as here the slope of the amplitudes with G is steepest.

The strongest effect of a changed maximum grazing rate on resilience we observe for slower predator grazing (Fig. B13). Here, if only the biomasses are perturbed, the predator never drops below its extinction threshold, as slower grazing prevents excessive over-exploitation of the prey with subsequent strong predator decay. Accordingly, we find a much higher number of extinctions for faster predator grazing compared to our original, intermediate setting (Fig. B14). The patterns of elasticity after pulse perturbations to the state variables are not strongly affected by changed predator grazing rates (Fig. B13, B14).

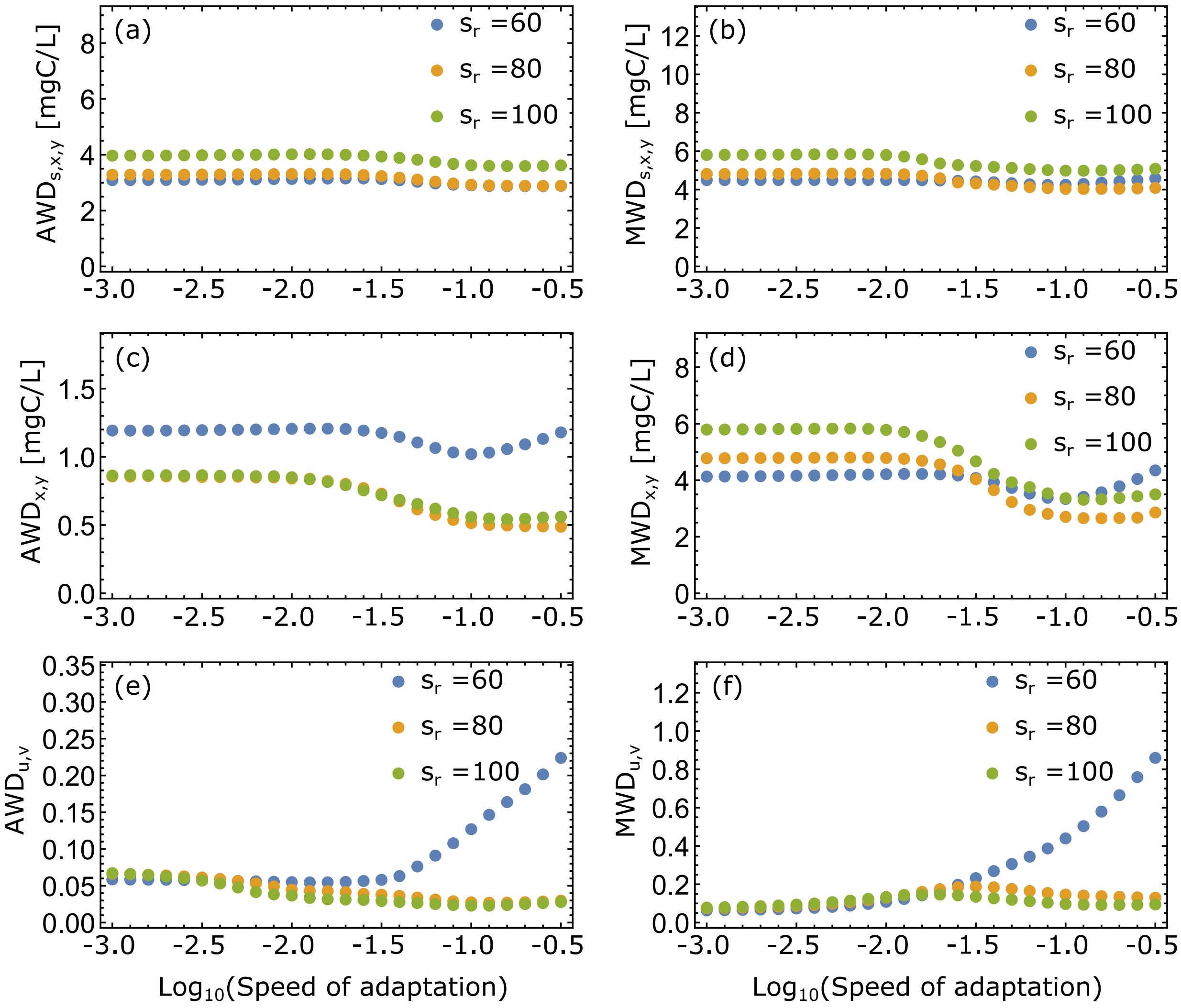

Figure B9 Resistance of dynamics in response to environmental press perturbations acting on the inflow concentration s_I for slower maximum predator grazing, implying a lower attack rate $a \approx 0.81\,\mathrm{mgC}^{-1}\,\mathrm{d}^{-1}$ and larger handling time $h \approx 0.52\,\mathrm{d}$. Further plot specifics are identical to Fig. 4.

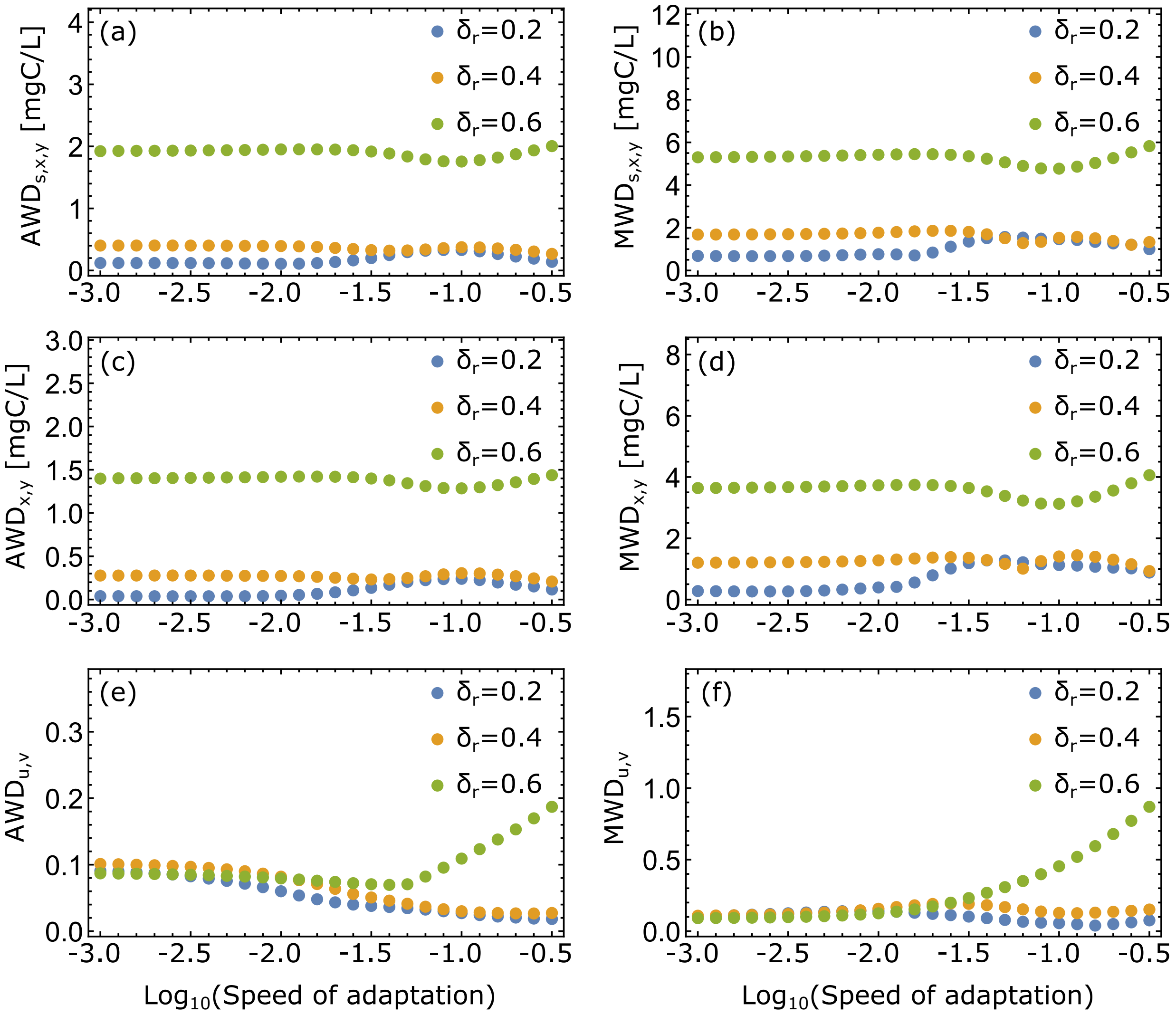

Figure B10 Resistance of dynamics in response to environmental press perturbations acting on the dilution rate δ for slower maximum predator grazing, implying a lower attack rate $a \approx 0.81\,\mathrm{mgC}^{-1}\,\mathrm{d}^{-1}$ and larger handling time $h \approx 0.52\,\mathrm{d}$. Further plot specifics are identical to Fig. 4.

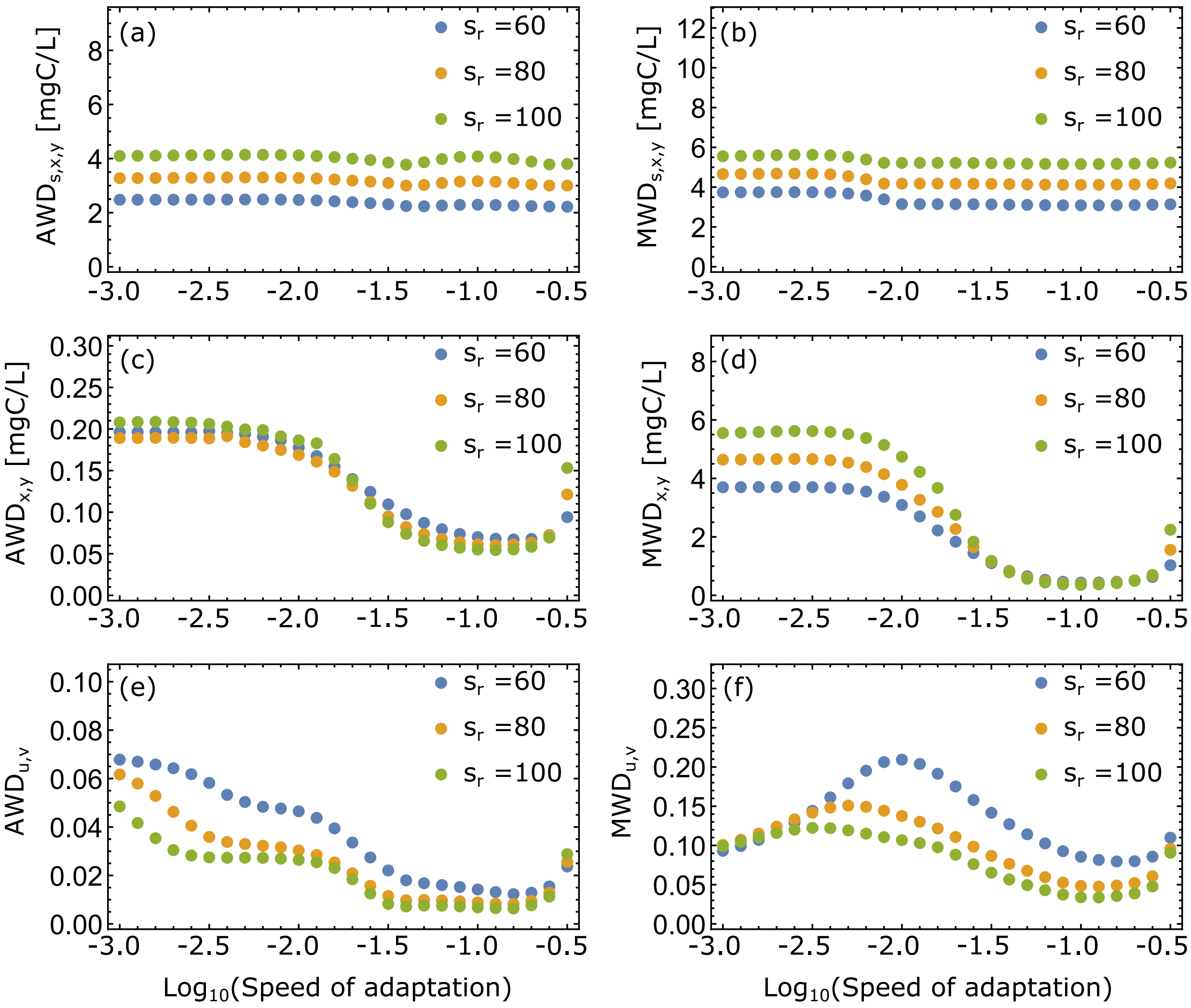

Figure B11 Resistance of dynamics in response to environmental press perturbations acting on the inflow concentration s_I for faster maximum predator grazing, implying a higher attack rate $a \approx 2.44\,\text{mgC}^{-1}\,\text{d}^{-1}$ and smaller handling time $h \approx 0.18\,\text{d}$. Further plot specifics are identical to Fig. 4.

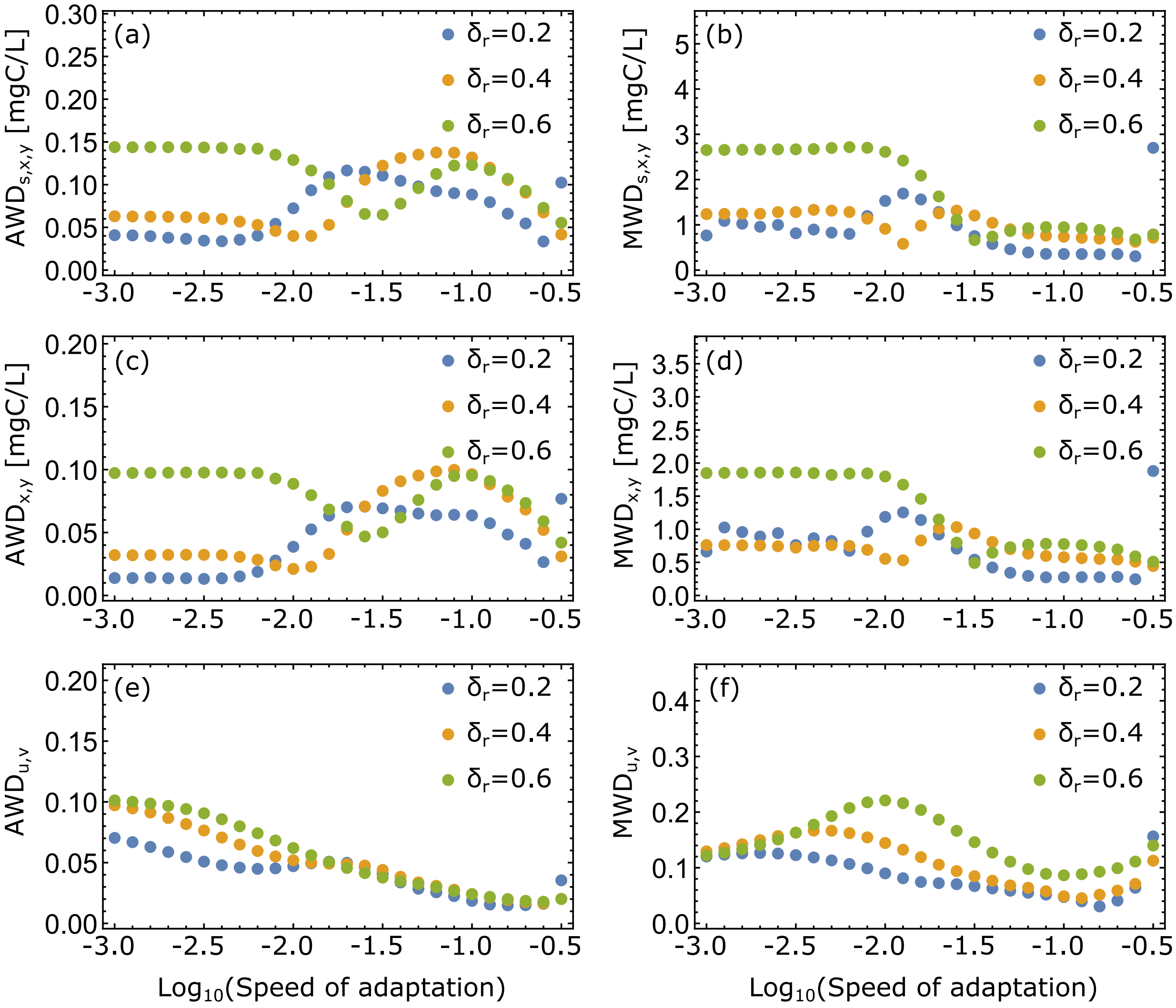

Figure B12 Resistance of dynamics in response to environmental press perturbations acting on the dilution rate δ for faster maximum predator grazing, implying a higher attack rate $a \approx 2.44\,\mathrm{mgC}^{-1}\,\mathrm{d}^{-1}$ and smaller handling time $h \approx 0.18\,\mathrm{d}$. Further plot specifics are identical to Fig. 4.

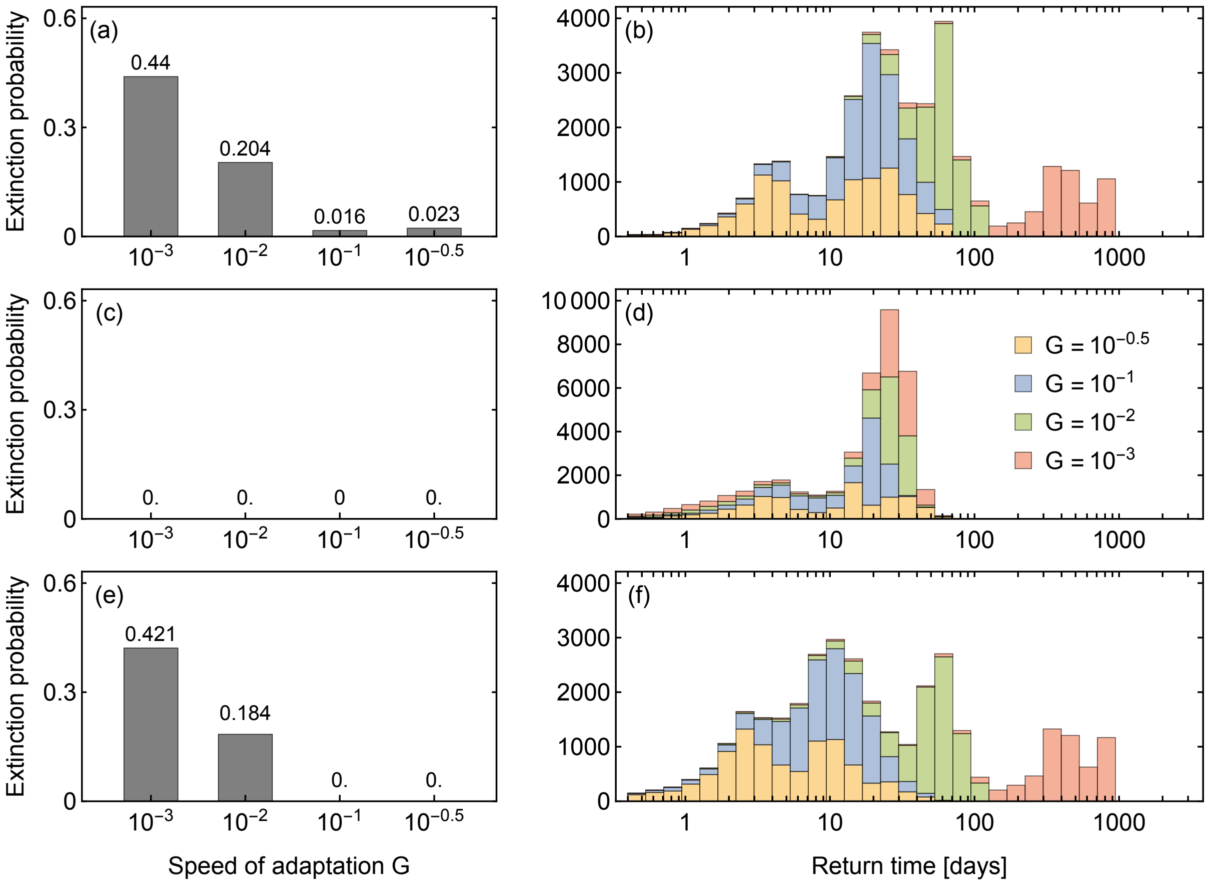

Figure B13 Resilience and elasticity of the predator-prey dynamics in response to random pulse perturbations for slower maximum predator grazing, implying a lower attack rate $a \approx 0.81\,\mathrm{mgC}^{-1}\,\mathrm{d}^{-1}$ and larger handling time $h \approx 0.52\,\mathrm{d}$. Further plot specifics are identical to Fig. 6.

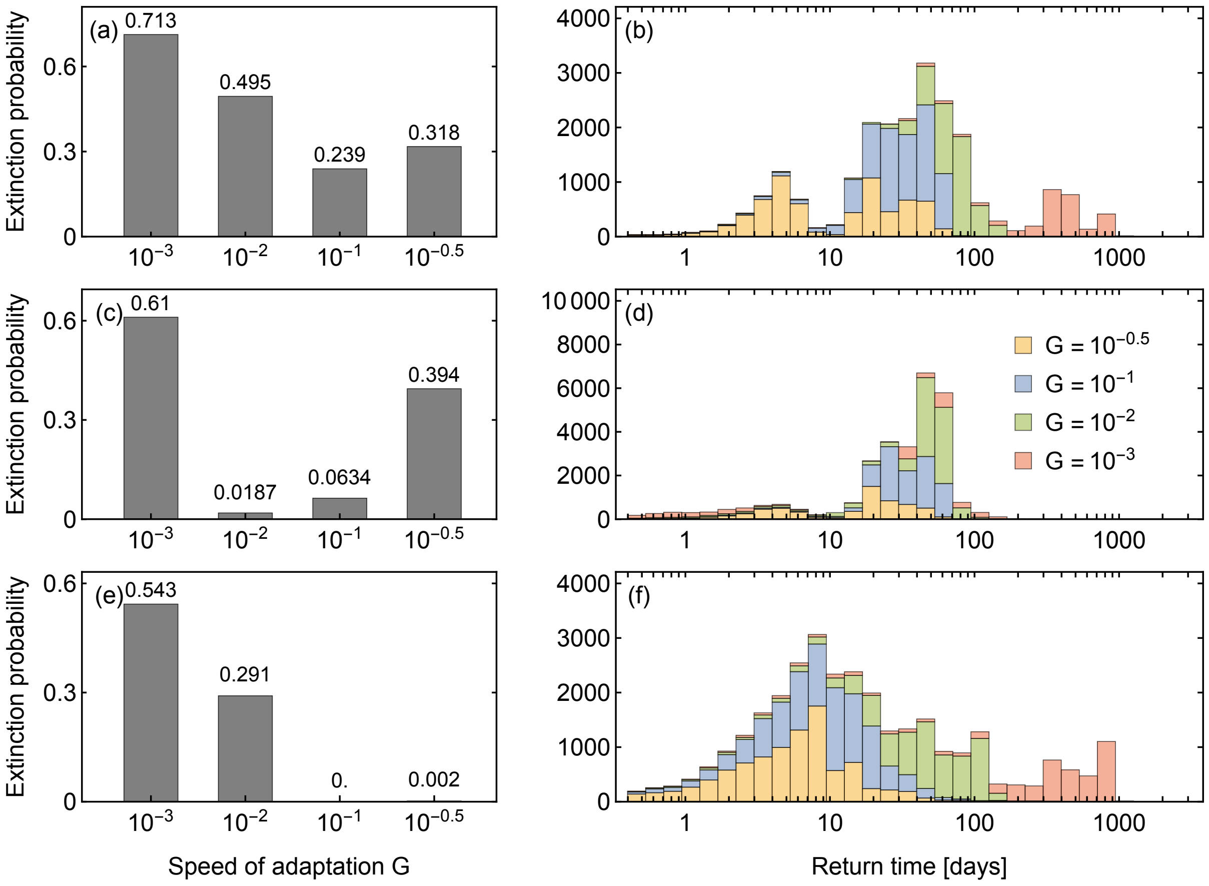

Figure B14 Resilience and elasticity of the predator-prey dynamics in response to random pulse perturbations for faster maximum predator grazing, implying a higher attack rate $a \approx 2.44\,\mathrm{mgC}^{-1}\,\mathrm{d}^{-1}$ and smaller handling time $h \approx 0.18\,\mathrm{d}$. Further plot specifics are identical to Fig. 6.
